# Comparative efficacy and safety of three internal fixation strategies for femoral neck fractures: a network meta-analysis

**DOI:** 10.3389/fmed.2026.1782357

**Published:** 2026-03-02

**Authors:** Min Liu, Liyan Mao, Chongyang Zhao, Huan Li, Tao Chen, Jialei Chen, Xiaobing Pu

**Affiliations:** 1 Department of Orthopedic Surgery, West China Hospital, Sichuan University, Chengdu, China; 2 West China School of Nursing, Sichuan University, Chengdu, China; 3 Department of Evidence-Based Medicine and Clinical Epidemiology, West China Hospital, Sichuan University, Chengdu, China; 4 West China School of Public Health and West China Fourth Hospital, Sichuan University, Chengdu, China; 5 Trauma Center, West China Hospital, Sichuan University, Chengdu, China; 6 Department of Orthopedic Surgery, West China Fourth Hospital, Sichuan University, Chengdu, China

**Keywords:** cannulated compression screws, dynamic hip screws, femoral neck system, femoral neck fracture, internal fixation

## Abstract

**Background:**

Currently, the most common internal fixation devices for femoral neck fractures are cannulated compression screws (CCS), dynamic hip screws (DHS), and femoral neck systems (FNS). However, no clear consensus exists regarding which device offers superior clinical efficacy and safety.

**Methods:**

We systematically searched three databases (PubMed, Embase, and Cochrane Library) for clinical studies published from their inception until March 12, 2025. We included studies that directly compared the three internal fixation methods: FNS, DHS, and CCS. Outcomes of interest were analyzed using pairwise and network meta-analyses.

**Results:**

This network meta-analysis included 23 studies comprising 55,910 patients. FNS demonstrated a statistically significant higher Harris Hip Score (MD 3.79, 95% CI 1.44–6.13) and a shorter fracture healing time (MD −1.00 months, 95% CI −1.53 to −0.48) compared to CCS. Both FNS and CCS were associated with lower rates of femoral head necrosis than DHS. CCS was superior to both FNS and DHS, showing significantly less intraoperative blood loss.

**Conclusion:**

This network meta-analysis indicates that FNS may have advantages over CCS and DHS in fracture healing time and reduced risk of femoral head necrosis. Regarding Harris Hip Score, FNS was statistically superior to CCS, but the improvement did not reach the minimal clinically important difference, suggesting limited clinical meaningfulness. However, its definitive clinical superiority and optimal indications remain uncertain, necessitating further high-quality studies to validate its clinical value and guide practice.

**Systematic review registration:**

https://www.crd.york.ac.uk/prospero/display_record.php?ID=CRD420251014243, identifier CRD420251014243.

## Introduction

Femoral neck fractures account for 3.6% of all fractures and 53% of hip fractures (1). With population aging, the incidence of hip fractures in older adults is projected to rise significantly (2). By 2050, the age-standardized incidence rate is expected to reach 1,055.30 cases per 100,000 person-years, imposing substantial socioeconomic and healthcare system burdens (3, 4). Moreover, femoral neck fractures in the elderly are predominantly osteoporotic fragility fractures, where intrinsically compromised bone quality and abnormal bone metabolism substantially increase the complexity of internal fixation and present an ongoing challenge for clinical decision-making (5).

Current surgical treatments for femoral neck fractures primarily involve internal fixation and hip arthroplasty (6). Among internal fixation methods, cannulated compression screws (CCS) and dynamic hip screws (DHS) are commonly used devices (7). The established CCS technique employs three screws arranged in an inverted triangular configuration, offering advantages such as minimal invasiveness, fracture compression, and rotational stability (8). This approach is particularly suitable for non-displaced fractures in elderly patients (9). However, CCS demonstrates limited shear resistance and provides only static compression, which may lead to micromotion at the fracture site during weight-bearing (10). This can hinder healing and increase risks of complications, such as screw cut-out and fixation failure. In contrast, DHS provides angular stability with enhanced lateral cortical support (11, 12). Its sliding compression mechanism promotes fracture healing, but the bulky design requires more extensive surgical exposure (13, 14). Consequently, the use of DHS may be restricted in patients with compromised overall health.

The femoral neck system (FNS; DePuy Synthes, Zuchwil, Switzerland), introduced in 2017, was designed to optimize minimally invasive techniques and enhance biomechanical stability (15, 16). The biomechanical study by Stoffel et al. demonstrated that the stability of FNS is superior to CCS and comparable to DHS (17). However, a clinical consensus is lacking regarding the comparative safety and efficacy of these three internal fixation techniques. Existing research remains confined to pairwise comparisons with limited sample sizes, resulting in reduced statistical power and imprecise effect estimates (18–21). To address this evidence gap, we conducted the first network meta-analysis integrating both direct and indirect evidence across all three fixation methods. This approach allows simultaneous comparison among multiple interventions, overcoming the limitations of previous pairwise analyses. By synthesizing a broader range of studies than previously available, we have strengthened the robustness of the pooled estimates and, through the unique advantages of network meta-analysis, provided a preliminary, probability-based ranking of treatments based on the current body of evidence.

This network meta-analysis was conducted to evaluate the comparative effectiveness and safety of the three fixation methods—FNS, CCS, and DHS. Our primary research question was: In patients with femoral neck fractures, which internal fixation method demonstrates superior clinical efficacy (as measured by the Harris Hip Score) and a more favorable safety profile, particularly regarding the risks of femoral neck shortening, femoral head necrosis, implant failure/cut-out, and fracture nonunion/delayed union?

The article is organized into the following sections. The Methods section describes the search strategy, study selection criteria, data extraction, risk-of-bias assessment, and statistical synthesis. The Results section reports the characteristics of the included studies, the pooled results, and the treatment rankings. The Discussion section interprets the key findings in a clinical context and discusses the limitations of the evidence. Finally, the Conclusion section summarizes the principal conclusions and suggests directions for future research.

## Methods

This study followed a registered protocol with PROSPERO (registration number: CRD420251014243), with one amendment implemented during the screening phase (rationale documented in the registry), and adheres to the Preferred Reporting Items for meta-analysis (PRISMA) guidelines (22).

### Search strategy

We systematically searched PubMed, Embase, and Cochrane Library databases from inception through March 12, 2025, for relevant clinical studies. The search strategy incorporated key subject headings, including femoral neck fractures, femoral neck system, cannulated screws, and dynamic hip screws. Detailed search terms are provided in Supplementary Information 1.

### Eligibility criteria

We developed the inclusion criteria according to the PICOS principle. (1) Population: Adults > 18 years diagnosed with unilateral femoral neck fractures. (2) Intervention: patients were treated with FNS. (3) Comparison: Fixation using either three CCS or DHS. (4) Outcome: Studies reporting at least one of the following primary outcomes: Harris Hip Score (HHS) and postoperative complications [e.g., femoral head necrosis (FHN), femoral neck shortening, implant failure/cut-out (IFC), fracture nonunion/delayed union (FNDU)], with a minimum 6-month follow-up for data completeness. (5) Study Design: Randomized controlled trials (RCTs) and cohort studies. Exclusion Criteria: (1) Patients with polytrauma, pathological fractures, open fractures, or revision surgeries. (2) Studies without full-text availability, duplicate datasets, or non-English publications. (3) biomechanics or animal research, finite element analysis, meta-analysis, review articles, case reports, conference abstracts, comments, and letters to editors.

### Definition of interventions

Each internal fixation device was explicitly defined to ensure comparability: CCS: Three parallel cannulated screws placed in an inverted triangular configuration. DHS: A sliding hip screw system consisting of a lag screw and a side plate fixed to the lateral femoral cortex. This category includes DHS with or without an additional anti-rotation screw. FNS: The dedicated femoral neck fixation system (DePuy Synthes) comprising an intramedullary nail, a locking bolt, and anti-rotation screw. Studies employing hybrid techniques or non-standard variants were excluded.

### Data extraction and quality assessment

Two reviewers independently extracted data using a predefined form and cross-verified for consistency. Discrepancies were resolved through discussion with a third reviewer. Extracted parameters included: first author, country, publication year, study design, intervention, sample size, patient age, gender, follow-up duration, fracture classification, and outcome measures. Primary outcomes comprised HHS and postoperative complications (e.g., FHN, femoral neck shortening, IFC, and FNDU). Secondary outcomes included intraoperative blood loss (IBL), operative time (OT), and fracture healing time (FHT). For each outcome within a study, data from the longest available follow-up time were extracted are reported in months. In addition, when studies reported outcomes as medians with ranges or interquartile ranges instead of means and standard deviations, we converted these summary statistics using the validated “Mean and Standard Deviation Calculator” developed by Tong et al. (23), which applies methods described by Luo et al. (24) and Wan et al. (25).

We employed the Cochrane Tool to assess the risk of bias for the included randomized controlled trials (RCTs) (26). Furthermore, for cohort studies, we used the Risk of Bias in Non-randomized Studies—of Interventions (ROBINS-I) tool to evaluate bias across seven domains: Bias due to confounding, participant selection, intervention classification, departures from intended interventions, missing data, outcome measurement, and selection of the reported result (27).

### Data analysis

Data were analyzed using frequentist network meta-analysis with evidence network visualization, applying the random effects model (package netmeta, version 3.2–0) described by Rücker (28) and Rücker and Schwarzer (29), within the R 4.3.2 (R Foundation for Statistical Computing, Vienna, Austria). Tau-squared (τ^2^) statistic calculated via the DerSimonian-Laird random-effects model was used to estimate the variance in heterogeneity between studies. We used the *I*^2^ statistic and Cochran’s Q statistic to assess heterogeneity across the entire network, within-design heterogeneity, and between-design inconsistency. *I*^2^ values < 25% indicated low heterogeneity; *I*^2^ values > 50% denoted substantial heterogeneity. Statistical significance was defined as *p* < 0.05. For multi-arm trials (studies with more than two treatment groups), we accounted for within-study correlation by appropriately splitting the shared control group sample size, preventing double-counting and ensuring valid standard error estimation. Missing outcome data were handled using complete-case analysis, including only participants with available data. For dichotomous outcomes with rare events (zero cells in contingency tables), we applied a continuity correction of 0.5.

We assessed local inconsistency within the network meta-analysis with the node-splitting method and quantified the contributions of direct and indirect evidence. Potential publication bias was evaluated using comparison-adjusted funnel plots symmetry and Egger’s test (30, 31). Pairwise meta-analyses were performed for all direct comparisons of primary and secondary outcomes. Treatments were ranked using P-scores (29). For the primary outcomes, absolute effects were calculated based on a baseline risk estimated from the pooled event rate in the CCS control groups. Furthermore, we conducted a leave-one-out sensitivity analysis by iteratively excluding individual studies to evaluate the robustness of the pooled effect estimates. Finally, to explore potential sources of heterogeneity, we pre-specified the following factors as potential effect modifiers for transitivity assessment and clinical heterogeneity prior to analysis: fracture displacement (Garden classification), reduction quality, osteoporosis status, age, and follow-up duration. Based on these, subgroup analyses and a meta-regression were performed for highly heterogeneous outcomes, using the gemtc package (version 1.1-0). A CINeMA (Confidence in Network Meta-Analysis) assessment was performed to evaluate the certainty of evidence for all outcomes (32, 33), and overall confidence was rated as High, Moderate, Low, or Very low.

## Results

### Included study

**FIGURE 1:**
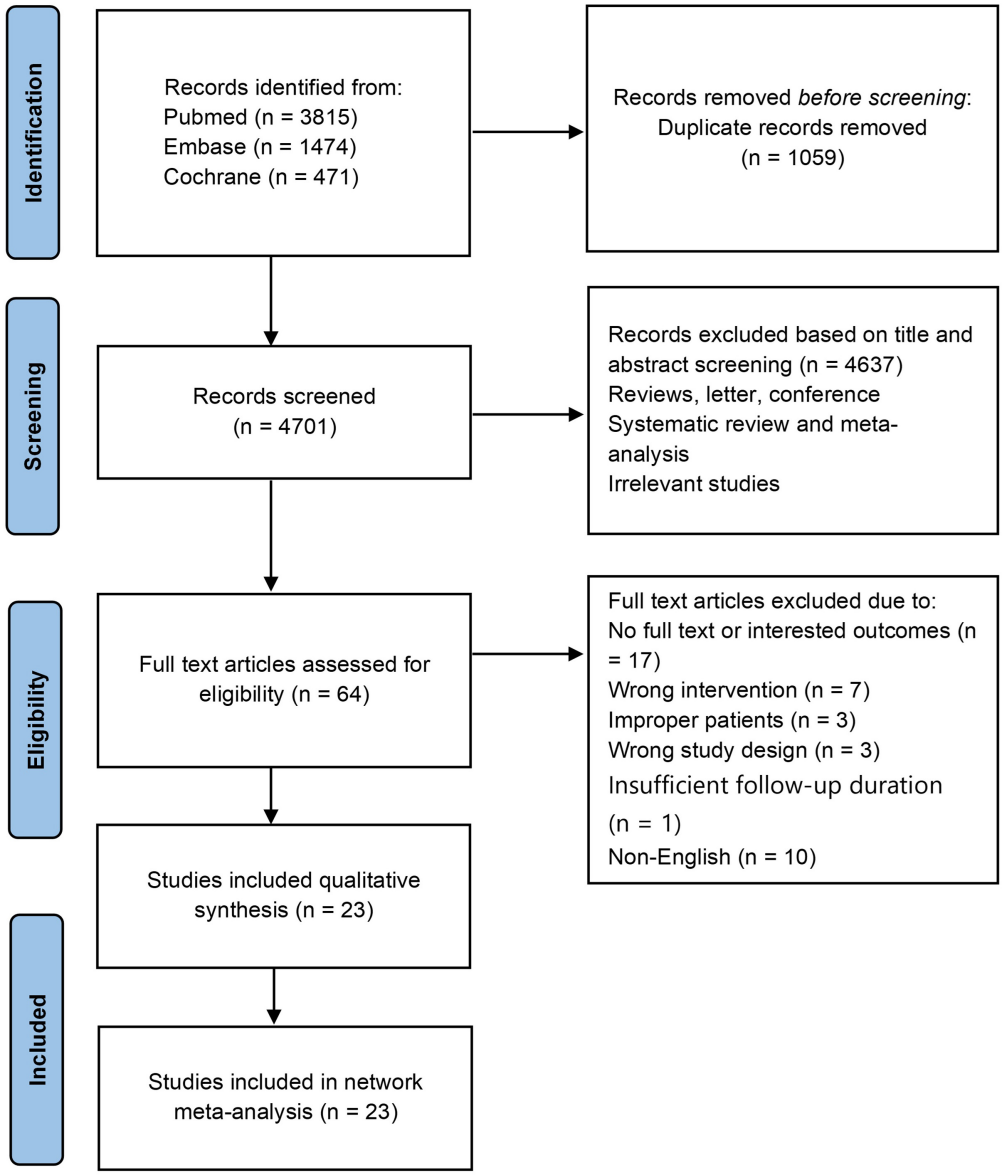
PRISMA flow diagram.

A comprehensive database search up to March 12, 2025, yielded 5,760 records. After removing 1,059 duplicates, 4,701 records underwent title and abstract screening. Of these, 4,637 records were excluded. Full-text assessment of the remaining 64 articles against predefined eligibility criteria resulted in the exclusion of 41 studies. Reasons for exclusion at the full-text stage are provided in Supplementary Table 1. Ultimately, 23 studies qualified for inclusion in the systematic review and network meta-analysis (Figure 1).

### Study characteristics and quality assessment

**FIGURE 2:**
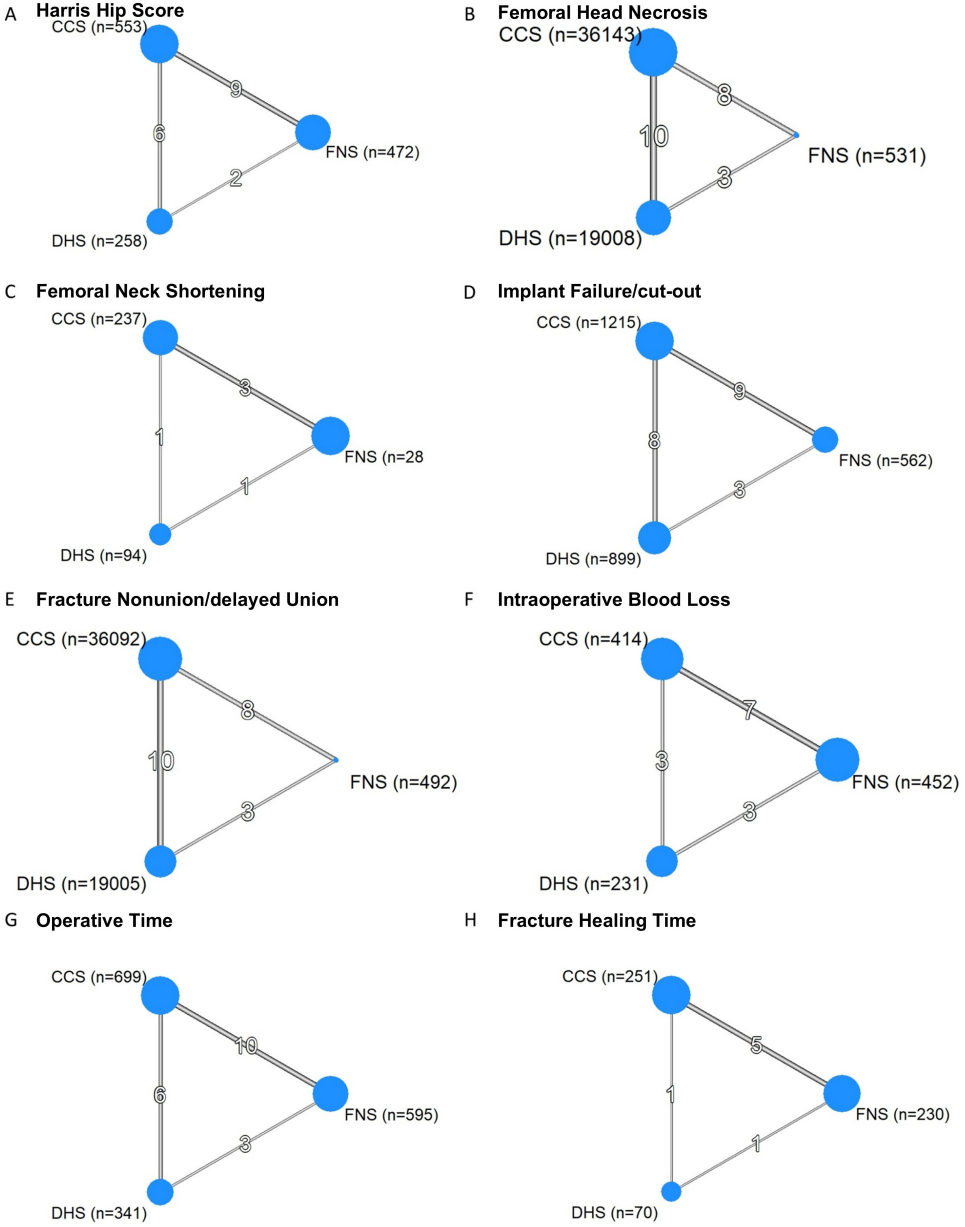
Network graphs of primary and secondary outcomes. Panels show network evidence diagrams for each outcome, with node size proportional to the number of participants receiving each treatment and edge thickness proportional to the number of studies informing each direct comparison. **(A)** Harris hip score. **(B)** Femoral head necrosis. **(C)** Femoral neck shortening. **(D)** Implant failure/cut-out. **(E)** Fracture nonunion/delayed union. **(F)** Intraoperative blood loss. **(G)** Operative time. **(H)** Fracture healing time. CCS, cannulated compression screw; DHS, dynamic hip screw; FNS, femoral neck system.

The analysis included 5 RCTs (34–38) and 18 cohort studies (39–56), encompassing 55,910 patients. Detailed study characteristics are provided in Supplementary Information 2. Figure 2 presents network evidence diagrams for all outcome measures. Regarding quality assessment, Supplementary Figure 1 illustrates the risk of bias for included RCTs. Notably, all RCTs showed limitations in blinding procedures. For cohort studies, ROBINS-I assessment revealed that most studies were judged to be at “Moderate” overall risk of bias, predominantly due to residual confounding, outcome assessment, and potential selective reporting inherent to retrospective designs. One study (40) was rated at “Serious” overall risk due to critical unmeasured confounding (e.g., fracture displacement not controlled). The full ROBINS-I assessment is summarized in Supplementary Table 2.

### Primary outcomes

#### Harris hip score

**FIGURE 3:**
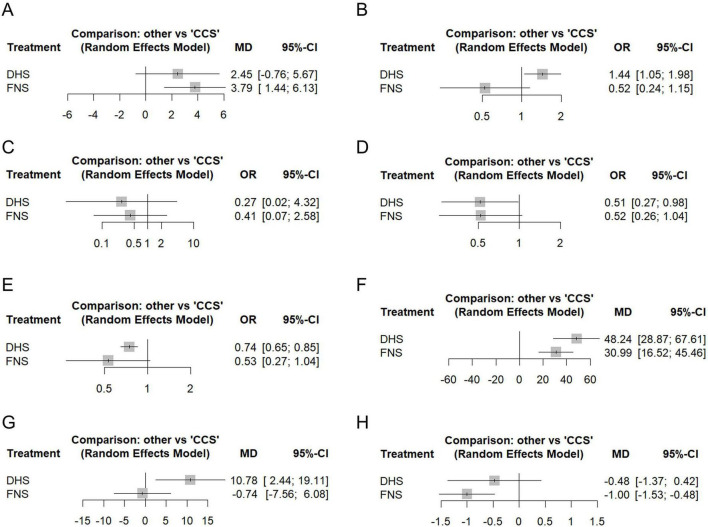
Forest plots for all outcomes from the network meta-analysis. Mean difference (MD) or odds ratio (OR) with 95% confidence intervals (CI) was used to measure the relative efficacy of different treatments compared to CCS. **(A)** Harris hip score. **(B)** Femoral head necrosis. **(C)** Femoral neck shortening. **(D)** Implant failure/cut-out. **(E)** Fracture nonunion/delayed union. **(F)** Intraoperative blood loss. **(G)** Operative time. **(H)** Fracture healing time. CCS, Cannulated compression screw; DHS, Dynamic hip screw; FNS, Femoral neck system.

The Harris Hip Score (HHS), a validated instrument assessing pain, function, deformity, and range of motion, served as a key indicator of hip joint function, with higher scores indicating better outcomes (57). HHS was reported by 15 studies comprising 1,283 patients (Supplementary Figure 2). Compared with CCS, FNS demonstrated significantly higher HHS scores (MD 3.79, 95% CI 1.44–6.13). No statistically significant differences were observed between DHS and CCS (MD 2.45, 95% CI −0.76 to 5.67) or between DHS and FNS (MD −1.33, 95% CI −4.86 to 2.19) (Figure 3 and Supplementary Figure 3). Substantial network heterogeneity was detected for this outcome (*I*^2^ = 90.1%).

#### Femoral head necrosis

FHN incidence was evaluated across 21 studies involving 55,682 patients (Supplementary Figure 4). The DHS group demonstrated significantly higher FHN risk than both the CCS group (OR 1.44, 95% CI 1.05–1.98) and the FNS group (OR 2.74, 95% CI 1.22–6.16), with no observed heterogeneity (*I*^2^ = 0%). Based on the CCS baseline risk of 5.45%, the absolute risk increase with DHS versus CCS was 2.21% (95% CI 0.26– 4.80%). No statistically significant difference emerged between FNS and CCS groups (OR 0.52, 95% CI 0.24–1.15) (Figure 3 and Supplementary Figure 5).

#### Femoral neck shortening

Femoral neck shortening, a common complication after internal fixation, was evaluated with a focus on clinically severe shortening (typically defined as > 10 mm), which is associated with impaired hip mechanics and function (58). It was evaluated in 5 studies comprising 612 patients (Supplementary Figure 6). Compared to CCS, neither DHS (OR 0.27, 95% CI 0.02–4.32) nor FNS (OR 0.41, 95% CI 0.07–2.58) showed significant differences in femoral neck shortening risk. Similarly, no significant difference emerged between DHS and FNS groups (OR 0.65, 95% CI 0.04–10.25) (Figure 3 and Supplementary Figure 7). Based on the CCS baseline risk of 12.86%, DHS and FNS were associated with absolute risk reductions (ARR) compared with CCS: −9.03% (95% CI −12.56 to 26.07) and −7.15% (95% CI −11.84 to 14.71), respectively. Substantial network heterogeneity was detected for this outcome (*I*^2^ = 82.2%).

#### Implant failure/cut-out

IFC was evaluated in 18 studies comprising 2,676 patients (Supplementary Figure 8). Compared with CCS, DHS significantly reduced the risk of IFC (OR 0.51, 95% CI 0.27–0.98), yielding an ARR of 3.40% (95% CI 0.13–5.15) based on the 7.21% baseline risk. While no significant difference was observed for FNS versus CCS (OR 0.52, 95% CI 0.26–1.04). DHS and FNS showed comparable IFC risk (OR 0.99, 95% CI 0.42–2.30) (Figure 3 and Supplementary Figure 9). All comparisons exhibited low heterogeneity (*I*^2^ = 23%).

#### Fracture nonunion/delayed union

FNDU was evaluated in 19 studies comprising 55,589 patients (Figure 3 and Supplementary Figure 10). Compared to CCS, DHS demonstrated significantly lower FNDU risk (OR 0.74, 95% CI 0.65–0.85). Based on the CCS baseline risk of 5.81%, this corresponds to an ARR of 1.44% (95% CI 0.83–1.95) for DHS. In contrast, no statistically significant difference was observed between FNS and CCS (OR 0.53, 95% CI 0.27–1.04) or between DHS and FNS (OR 1.41, 95% CI 0.71–2.77) (Figure 3 and Supplementary Figure 11). All comparisons exhibited minimal heterogeneity (*I*^2^ = 0%).

### Secondary outcomes

#### Intraoperative blood loss

IBL was recorded in 11 studies comprising 1,097 patients (Figure 3 and Supplementary Figure 12). Both DHS (MD 48.24 mL, 95% CI 28.87–67.61) and FNS (MD 30.99 mL, 95% CI 16.52–45.46) demonstrated significantly greater blood loss than CCS. No significant difference was observed between DHS and FNS (MD 17.25 mL, 95% CI −1.80 to 36.30) (Figure 3 and Supplementary Figure 13). All comparisons exhibited substantial heterogeneity (*I*^2^ = 96.8%).

#### Operative time

OT was recorded in 17 studies comprising 1,635 patients (Supplementary Figure 14). DHS procedures were significantly longer than both CCS (MD 10.78 min, 95% CI 2.44–19.11) and FNS (MD 11.52 min, 95% CI 2.34–20.70). No significant difference emerged between FNS and CCS (MD −0.74 min, 95% CI −7.56 to 6.08) (Figure 3 and Supplementary Figure 15). All comparisons exhibited substantial heterogeneity (*I*^2^ = 94.5%).

#### Fracture healing time

FHT was evaluated across 7 studies involving 551 patients (Supplementary Figure 16). FNS demonstrated significantly shorter healing time than CCS (MD −1.00 months, 95% CI −1.53 to −0.48). No significant differences were observed between DHS and FNS (MD 0.53 months, 95% CI −0.38 to 1.43) or between DHS and CCS (MD −0.48 months, 95% CI −1.37 to 0.42) (Figure 3 and Supplementary Figure 17). All comparisons exhibited substantial heterogeneity (*I*^2^ = 94.2%).

#### Ranking of treatments

**FIGURE 4:**
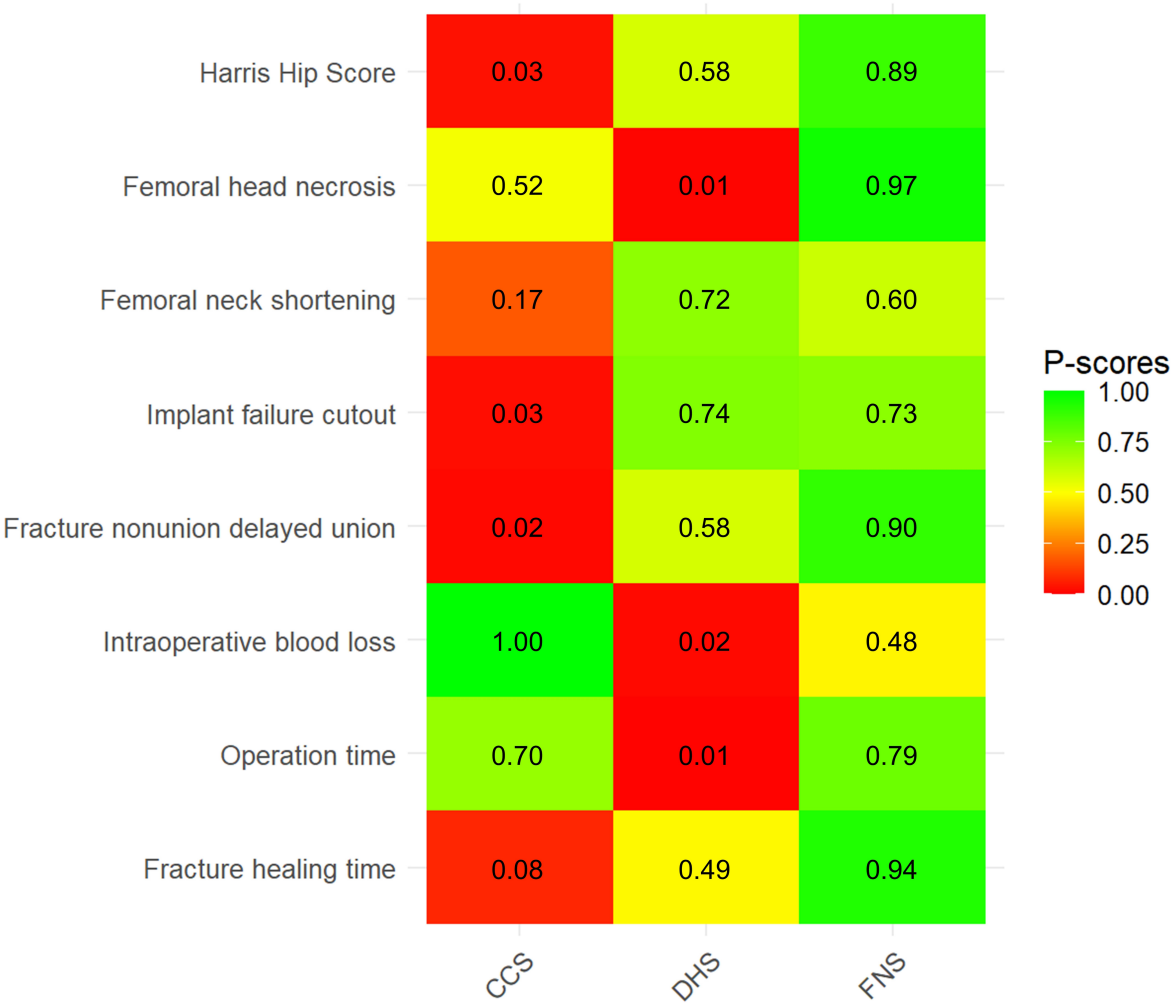
P-score ranking for primary and secondary outcomes. Color intensity corresponds to P-Score values (darker green = higher rank probability). P-score quantifies the probability of a treatment being ranked first (range: 0–1). Numeric values indicate P-scores. CCS, Cannulated compression screw; DHS, Dynamic hip screw; FNS, Femoral neck system.

Figure 4 presents the P-score rankings for the three fixation technologies. FNS ranked first for HHS, FHN, FNDU, OT, and FHT, while securing second position across all remaining outcomes. CCS demonstrated a superior ranking only for IBL. DHS showed marginally higher P-scores than FNS in femoral neck shortening and IFC, earning first rank for these outcomes.

#### Publication bias and inconsistency

Comparison-adjusted funnel plots for all outcomes demonstrated symmetrical distributions (Supplementary Figure 18), with Egger’s test *p*-values > 0.05 for outcomes with ≥ 10 studies, suggesting no significant publication bias. Egger’s test was not performed for femoral neck shortening and FHT due to limited studies ( < 10). Global inconsistency, assessed via the design-by-treatment interaction model, was statistically significant for HHS (Q = 11.31, *p* = 0.010), IBL (Q = 91.03, *p* < 0.001), OT (Q = 60.99, *p* < 0.001), and FHT (Q = 13.64, *p* = 0.0002), but not for other outcomes (all *p* > 0.05, see Supplementary Information 1 for details). Local inconsistency testing using the node-splitting approach revealed significant disagreement between direct and indirect evidence for IBL in the FNS vs. CCS (*p* = 0.002) and DHS vs. FNS (*p* = 0.014) comparisons. For all other outcomes, no significant local inconsistency was detected (all *p* > 0.05; see Supplementary Figures 19–26 for full results).

#### Sensitivity and exploratory analyses

Sensitivity analyses demonstrated robust findings for most outcomes upon sequential exclusion of individual studies (Supplementary Figures 27–32). For femoral head necrosis, exclusion of the study by Jetto et al. (40)—the largest retrospective registry study contributing substantial weight—resulted in a slight attenuation of the effect estimate for DHS versus CCS, with the confidence interval crossing unity (from OR 1.44, 95% CI 1.05–1.98 to OR 1.39, 95% CI 0.96–2.02). However, the direction of effect remained consistent, and the estimates for other comparisons (FNS vs. CCS and DHS vs. FNS) were largely unchanged. For fracture nonunion/delayed union, exclusion of the same study rendered the DHS versus CCS comparison non-significant, with substantially widened confidence intervals (OR changed from 0.74, 95% CI 0.65–0.85 to 0.72, 95% CI 0.49–1.06), while other comparisons remained stable, indicating its notable influence on outcome stability. Sensitivity analysis was not feasible for femoral neck shortening and FHT due to the limited included studies, where the removal of any single trial disrupted network connectivity.

Subgroup analyses revealed that in the fracture type subgroup, the DHS versus CCS comparison became statistically significant, unlike in the overall analysis, and heterogeneity decreased from *I*^2^ = 90.1–70.9% (detailed in Supplementary Table 3). In contrast, no significant differences from the primary treatment effects or substantial reductions in heterogeneity were observed in the subgroups based on study design or follow-up duration. For femoral head necrosis, the subgroup analysis by DHS design showed an OR of 1.46 (95% CI 0.84–2.54) for standard DHS vs. CCS, and an OR of 0.96 (95% CI 0.38–2.39) for DHS with an anti-rotation screw vs. CCS.

Network meta-regression revealed that age had a consistent negative moderating effect across all outcomes. However, this effect was statistically significant only for intraoperative blood loss (as shown in Supplementary Table 4). The relative treatment effects and their statistical significance for all comparisons remained consistent with the primary analysis.

#### Assessment of the certainty of evidence

The certainty of evidence, assessed using the CINeMA framework, was low or very low for the majority of comparisons across outcomes. The most common reasons for downgrading were within-study bias, imprecision, or heterogeneity. Detailed ratings for each outcome are presented in Supplementary Table 5.

## Discussion

This network meta-analysis was conducted to evaluate the comparative effectiveness and safety of FNS, CCS, and DHS for femoral neck fractures, with the aim of determining the optimal fixation strategy. Our findings, within the constraints of the available data, indicate that no single method was universally superior across all outcomes, but each demonstrated distinct strengths: FNS significantly outperformed CCS in HHS and fracture healing time, while also significantly surpassing DHS in operative time and femoral head necrosis. DHS showed significantly lower risks of implant failure/cut-out and fracture nonunion/delayed union compared to CCS, with comparable performance to FNS. CCS demonstrated significant superiority over DHS in femoral head necrosis and operative time, while also showing a significant reduction in intraoperative blood loss compared to both DHS and FNS. However, given that the CINeMA assessment indicated generally low or very low certainty of evidence for most comparisons, the P-score rankings which offer a probability-based hierarchy of relative treatment performance should not be equated with definitive clinical superiority, especially for outcomes with small, non-significant, or highly heterogeneous between-treatment differences. Therefore, clinical decision-making should prioritize specific effect estimates, confidence intervals, and patient-centered goals over the rank order itself.

Our network meta-analysis found that FNS yielded significantly higher HHS compared to CCS, aligning with findings from Jiang et al. and Patel et al. (18, 59). The superior HHS observed with the FNS may be attributed to its capacity to prevent femoral neck shortening while mitigating complications, including femoral head necrosis and implant failure/cut-out. Substantial evidence indicates that femoral neck shortening markedly compromises hip function and quality of life. Mechanistically, this results from reduced moment arms of hip abductor muscles, causing limping and weakened abduction strength that collectively impair hip function (60). Furthermore, femoral head necrosis and fixation failure induce hip pain and restricted motion at varying severity levels, thereby directly diminishing HHS outcomes (61, 62). While the FNS showed a statistically significant improvement in HHS over CCS (mean difference 3.79), this change remains below the reported Minimal Clinically Important Difference (MCID) of 6–10 points for HHS in this population (63–65). Although the result suggests a positive trend, its clinical relevance at the individual level is likely modest. Therefore, when choosing a treatment, this potential functional benefit of FNS should be weighed against its complication risks, cost, and technical demands. The considerable heterogeneity observed in HHS outcomes underscores the potential role of fracture type as an effect modifier, as suggested by our subgroup analysis of mixed populations (Garden I-IV/II-IV). Unfortunately, the available literature’s lack of outcome stratification by fracture subtype prevented a more granular analysis. Consequently, our findings should be interpreted with caution. Future high-quality research focusing on specific fracture types is essential to definitively elucidate the comparative effectiveness of these three fixation devices on HHS, providing tailored guidance for clinical implant selection.

For femoral neck shortening, although no statistically significant differences were observed among the three fixation techniques, the odds ratios suggested a potential hierarchy: DHS demonstrated marginally superior outcomes relative to FNS, with CCS showing the least favorable results. It is important to note that the considerable heterogeneity and the sparse, inconsistent high-quality evidence necessitate a cautious interpretation of these results. Evidence suggests that fracture type (Garden III–IV) and reduction quality (III–IV) are key predictors of postoperative femoral neck shortening, potentially explaining the significant heterogeneity in our findings (66–68). Demographic variations may represent another source of heterogeneity. Older patients with osteoporosis frequently demonstrate compromised bone quality, prolonged fracture healing, and poorer implant stability. These factors increase susceptibility to deformation during weight-bearing, consequently leading to femoral neck shortening (69, 70). Unfortunately, the limited number of available studies precluded meaningful subgroup analysis. Further studies are necessary.

The significantly higher incidence of femoral head necrosis observed with DHS compared to CCS and FNS stems from its larger bolt diameter. This design characteristic generates greater surgical trauma, necessitates more bone removal, and induces rotational displacement during insertion—all factors contributing to impaired femoral head perfusion (13, 71, 72). In contrast, FNS’s smooth nail design and minimally invasive approach preserve blood supply. Furthermore, DHS demonstrated superior outcomes versus CCS for both implant failure/cut-out and fracture nonunion/delayed union. DHS converts vertical loads into axial compression at the fracture site via a sliding compression mechanism, enhancing interfragmentary compression and promoting fracture healing (73). Additionally, its larger lag screw diameter also increases bone-implant contact area and friction, reducing the risks of implant failure/cut-out and fracture nonunion/delayed union. FNS exhibited mechanical stability comparable to DHS with similar compression advantages (74, 75). Consequently, no significant differences were observed between FNS and DHS regarding implant failure/cut-out, fracture nonunion/delayed union, and fracture healing time. It should be noted that the pooled estimates for femoral head necrosis and fracture nonunion/delayed union were influenced by a single large retrospective registry study (Jetto et al.), which contributed substantial weight to these analyses. Sensitivity analysis excluding this study attenuated the DHS versus CCS comparisons for both outcomes to non-significance. However, the direction of effect remained consistent across both outcomes, and comparisons involving FNS were largely unchanged. These findings suggest that while the precision and statistical significance of the DHS versus CCS comparisons may be overestimated by the inclusion of this large study, the overall trends remain mechanistically plausible. Nevertheless, confirmation in future high-quality studies is warranted.

CCS demonstrated the lowest intraoperative blood loss, followed by FNS, with DHS exhibiting the highest volumes. Operative time was the longest for DHS, while FNS procedures were non-significantly shorter than CCS. Notably, FNS achieved this efficiency despite surgeons’ relative inexperience with this newer system, suggesting that it may offer greater ease of use compared to the established CCS and DHS techniques. The substantial heterogeneity observed likely stems from variations in patient age, which our meta-regression indicated was a significant moderator for blood loss, with the differences between treatments diminishing in older patients.

Notably, caution is required when extrapolating our findings to older adults. Although our study included adults across the age spectrum, subgroup analyses by age or osteoporosis status were not feasible. Since femoral neck fractures in the elderly are predominantly osteoporotic, their distinct bone-healing biology—such as impaired osteoblast function, aberrant stem cell activity, and a pro-resorptive microenvironment—may alter the healing response to different fixation devices (76, 77). Simultaneously, the degraded biomechanical environment, characterized by reduced bone mineral density and loss of trabecular connectivity, can directly compromise implant stability (78–80). Consequently, in osteoporotic bone, implant fixation strength, cut-out resistance, and control of micro-motion become paramount. Therefore, our primary findings may not directly inform decision-making for older osteoporotic patients, underscoring the urgent need for high-quality, targeted studies in this population. Moreover, the interpretation of these pooled estimates requires caution due to substantial statistical heterogeneity. Several outcomes in this analysis, including HHS, intraoperative blood loss, and fracture healing time, exhibited substantial statistical heterogeneity (*I*^2^ > 90%). As noted in the preceding discussions, potential sources are multifaceted and may include clinical diversity (e.g., variations in patient age, displacement, osteoporosis status, and rehabilitation protocols) and methodological diversity (e.g., differences in surgical technique, follow-up duration, and study design). Notably, the predominance of retrospective cohort studies in our analysis (18 of 23, mostly moderate risk of bias) introduces residual confounding from unmeasured factors—such as fracture displacement and surgeon selection bias—that could not be fully accounted for. These methodological limitations likely contribute substantially to the observed heterogeneity. While random-effects models were employed to incorporate this between-study variance, the high heterogeneity fundamentally suggests that the pooled effect estimates should be interpreted as an average across a diverse set of clinical scenarios rather than a precise predictor for all individuals. This underscores the need for caution when generalizing these findings and highlights that clinical decision-making should remain tailored to specific patient and contextual factors.

While the FNS has shown promising results for femoral neck fractures, several concerns require urgent resolution, such as the potential for subtrochanteric fracture, unclear cost-effectiveness, and a substantial lack of long-term evidence. Firstly, clinical case reports have suggested a potential risk of subtrochanteric fractures with the FNS (81, 82). This hypothesis is further corroborated by several biomechanical studies. Fan et al. demonstrated that the peak von Mises stress on the distal locking screw of the FNS in the lesser trochanteric region reaches approximately 80 MPa—higher than that observed with three CCS (83). Similarly, Jung et al. found that a longer lateral plate length and a valgus bolt position elevate cortical bone strain around the distal-most locking screw in FNS, consequently increasing the risk of subtrochanteric fracture (84). However, as the available evidence on FNS-related subtrochanteric fractures is largely confined to biomechanics and lacks clinical data, high-quality clinical studies are required to clarify the incidence and influencing factors of this complication. Secondly, cost-effectiveness plays an important role in clinical decisions. However, there is currently no cost-effectiveness analysis evidence demonstrating that the significantly higher price of FNS over three CCS can be offset by commensurate benefits (85). Finally, as a relatively new technology, FNS has a limited history of clinical application, and its optimal clinical indications-such as appropriate patient age and specific fracture types-remain undefined. A study initiated by the AO Foundation found that in younger patients under 58 years, HHS were uniformly good to excellent. In contrast, the distribution of HHS in patients aged 58–80 years was similar to that in those over 80 years, ranging widely from 25 to 100 (86). This indicates that postoperative functional recovery with FNS is superior in younger populations compared to older adults. This observation is consistent with the results of our meta-regression analysis, which demonstrated that the difference in postoperative functional outcomes between FNS and the other two internal fixation devices diminishes with increasing patient age. This attenuated benefit may be attributed to a reduction in the biomechanical stability provided by FNS in older patients particularly those with osteoporosis, which is even inferior to that of CCS (87). Consequently, the clinical application of FNS in elderly or osteoporotic patients should be cautious until high-quality RCTs are conducted to definitively establish its safety and efficacy in these specific populations. Furthermore, most existing studies are retrospective cohort analyses with relatively short follow-up periods, making it difficult to accurately assess the long-term incidence of complications such as femoral head necrosis and implant failure. Therefore, further high-quality, long-term studies are essential to evaluate the safety and efficacy of FNS and to define its optimal clinical applications.

Given the current evidence, clinical implant selection must be individualized based on their distinct efficacy-safety profiles. The FNS demonstrates advantages in promoting early function and healing, particularly for younger patients or those with displaced fractures where its biomechanical stability is most beneficial. Caution is advised for elderly or osteoporotic patients pending long-term safety data. The DHS provides reliable mechanical stability with a lower risk of implant failure and nonunion compared to CCS, making it a robust choice for unstable fracture patterns. However, its greater risk of femoral head necrosis necessitates careful consideration, especially in subcapital or transcervical fractures. The CCS remains the standard for minimally invasive fixation of non-displaced fractures, particularly when minimizing surgical trauma and blood loss is paramount. These recommendations are tempered by critical evidence gaps that must be addressed in future research: (1) long-term comparative safety, especially regarding subtrochanteric fracture risk with FNS and avascular necrosis across all implants; (2) stratified efficacy data for key but poorly reported modifiers, including precise osteoporosis status, fracture reduction quality, age, and specific displacement patterns; and (3) formal cost-effectiveness analyses. Until such high-quality, granular evidence emerges, a cautious and patient-tailored approach to implant selection, guided by surgeon expertise, remains prudent.

### Limitations

This study has several limitations. First, despite a comprehensive literature screening, the final sample size remains limited, particularly regarding high-quality RCTs comparing FNS with the other two interventions. Future analyses should incorporate more robust evidence to enhance the reliability of conclusions. Second, broad inclusion criteria for age and fracture classification (Garden/Pauwels) were employed due to evidence constraints, which inevitably introduced confounding variables. Third, significant heterogeneity in certain outcomes precluded subgroup analysis of clinical modifiers (e.g., age, fracture type, reduction quality) due to insufficiently stratified primary data. Future research should prioritize larger RCTs with refined inclusion criteria and conduct stratified analyses to evaluate procedure efficacy across patient subgroups, thereby facilitating personalized clinical decision-making. Last, given that the CINeMA assessment indicated generally low or very low certainty of evidence for many comparisons, our findings should be interpreted cautiously, underscoring the need for larger, well-designed trials to strengthen the evidence base.

## Conclusion

This network meta-analysis demonstrates that FNS shows potential benefits over CCS and DHS in terms of fracture healing time and reduced risk of femoral head necrosis. Regarding HHS, FNS was statistically superior to CCS, but the magnitude of improvement did not reach the minimal clinically important difference, suggesting limited clinical meaningfulness. Several limitations remain, such as uncertain clinical indications, an unclear risk of subtrochanteric fracture, and the absence of high-quality, long-term follow-up studies. Therefore, until more high-level and robust evidence becomes available, clinicians should adopt a selective and judicious approach to its use. Future large-scale, prospective studies are essential to definitively determine its clinical superiority and establish guidelines for its optimal use.

## Data Availability

The raw data supporting the conclusions of this article will be made available by the authors, without undue reservation.

## References

[1] Lu H Shen H Zhou S Ni W Jiang D. Biomechanical analysis of the computer-assisted internal fixation of a femoral neck fracture. *Genes Dis.* (2019) 7:448–55. 10.1016/j.gendis.2019.04.006 32884999 PMC7452504

[2] Harris E Clement N MacLullich A Farrow L. The impact of an ageing population on future increases in hip fracture burden. *Bone Joint J.* (2024) 106-B:62–8. 10.1302/0301-620X.106B1.BJJ-2023-0740.R1 38160690

[3] Tian C Shi L Wang J Zhou J Rui C Yin Y Global, regional, and national burdens of hip fractures in elderly individuals from 1990 to 2021 and predictions up to 2050: a systematic analysis of the Global Burden of Disease Study 2021. *Arch Gerontol Geriatr.* (2025) 133:105832. 10.1016/j.archger.2025.105832 40112671

[4] Bhandari M Swiontkowski M. Management of acute hip fracture. *N Engl J Med.* (2017) 377:2053–62. 10.1056/NEJMcp1611090 29166235

[5] Mukhopadhaya J Bhadani JS. Fixation failure in osteoporotic bone: a review of complications and outcomes. *Indian J Orthop.* (2025) 59:389–404. 10.1007/s43465-024-01316-y 40201917 PMC11973034

[6] Zelle BA Salazar LM Howard SL Parikh K Pape HC. Surgical treatment options for femoral neck fractures in the elderly. *Int Orthop.* (2022) 46:1111–22. 10.1007/s00264-022-05314-3 35187589

[7] Liang C Cao Y Lin Z Liu G Zhang C Hu Y. Open reduction and internal fixation of irreducible displaced femoral neck fracture with femoral Neck System: a preliminary study. *BMC Musculoskelet Disord.* (2023) 24:826. 10.1186/s12891-023-06839-3 37858123 PMC10585802

[8] Yang JJ Lin LC Chao KH Chuang SY Wu CC Yeh TT Risk factors for nonunion in patients with intracapsular femoral neck fractures treated with three cannulated screws placed in either a triangle or an inverted triangle configuration. *J Bone Joint Surg Am.* (2013) 95:61–9. 10.2106/JBJS.K.01081 23283374

[9] Panteli M Rodham P Giannoudis PV. Biomechanical rationale for implant choices in femoral neck fracture fixation in the non-elderly. *Injury.* (2015) 46:445–52. 10.1016/j.injury.2014.12.031 25597514

[10] Basso T. Internal fixation of fragility fractures of the femoral neck. *Acta Orthop Suppl.* (2015) 86:1–36. 10.3109/17453674.2015.1056702 26036608

[11] Ma J Zhao Z Zhi X Wang H Wang W. Finite element comparative analysis of three different internal fixation methods in the treatment of Pauwels type III femoral neck fractures. *BMC Musculoskelet Disord.* (2022) 23:1030. 10.1186/s12891-022-06003-3 36447275 PMC9706946

[12] Augat P Bliven E Hackl S. Biomechanics of femoral neck fractures and implications for fixation. *J Orthop Trauma.* (2019) 33:S27–32. 10.1097/BOT.0000000000001365 30540669

[13] Shu DP Xiao YP Bei MJ Ji T Peng YJ Ma B Dynamic compression locking system versus multiple cannulated compression screw for the treatment of femoral neck fractures: a comparative study. *BMC Musculoskelet Disord.* (2020) 21:230. 10.1186/s12891-020-03259-5 32284062 PMC7155247

[14] Zhang LL Zhang Y Ma X Liu Y. Multiple cannulated screws vs. dynamic hip screws for femoral neck fractures: a meta-analysis. *Orthopade.* (2017) 46:954–62. 10.1007/s00132-017-3473-8 29022057

[15] Zhang X Zheng C Huang J Chen H Lei J Huang C. Comparison of three different internal fixation methods in the treatment of femoral neck fracture. *Heliyon.* (2024) 10:e34582. 10.1016/j.heliyon.2024.e34582 39149078 PMC11325052

[16] Lee HH Chun YS Kim WY Lim YW Kim SC. Comparison of femoral neck system fixation outcomes in nondisplaced femoral neck fractures: a multicenter retrospective study of patients aged below and above 75 years. *Eur J Trauma Emerg Surg.* (2025) 51:210. 10.1007/s00068-025-02891-x 40387972

[17] Stoffel K Zderic I Gras F Sommer C Eberli U Mueller D Biomechanical evaluation of the femoral neck system in unstable Pauwels III femoral neck fractures: a comparison with the dynamic hip screw and cannulated screws. *J Orthop Trauma.* (2017) 31:131–7. 10.1097/BOT.0000000000000739 27755333

[18] Jiang J Chen J Xing F Liu H Xiang Z. Comparison of femoral neck system versus cannulated screws for treatment of femoral neck fractures: a systematic review and meta-analysis. *BMC Musculoskelet Disord.* (2023) 24:285. 10.1186/s12891-023-06378-x 37055749 PMC10099821

[19] Zhuang K Wu J Yang Y Bai T Li B. Comparison of clinical efficacy between femoral neck system and cannulated screw in Pauwels type III femoral neck fracture: a meta-analysis. *J Back Musculoskelet Rehabil.* (2025) 38:71–82. 10.1177/10538127241296340 39970468

[20] Jiang QL Cao Y Bai X Deng Y Li Y. Femoral neck system versus dynamic hip screw for fixation of femoral neck fracture in the adult: a meta-analysis. *J Pak Med Assoc.* (2024) 74:335–40. 10.47391/JPMA.9556 38419236

[21] Zhang J Chang X Sun Z Tang X. Comparison of femoral neck system versus cannulated compression screws in treating femoral neck fractures: a systematic review and meta-analysis. *Asian J Surg.* (2023) 46:3259–60. 10.1016/j.asjsur.2023.03.013 36933962

[22] Hutton B Moher D Cameron C. The PRISMA extension statement. *Ann Intern Med.* (2015) 163:566–7. 10.7326/L15-5144-2 26436629

[23] Mean Variance Estimation Available online at: https://www.math.hkbu.edu.hk/${\sim}$tongt/papers/median2mean.html (accessed Feb 11, 2026) (2026).

[24] Luo D Wan X Liu J Tong T. Optimally estimating the sample mean from the sample size, median, mid-range, and/or mid-quartile range. *Stat Methods Med Res.* (2018) 27:1785–805. 10.1177/0962280216669183 27683581

[25] Wan X Wang W Liu J Tong T. Estimating the sample mean and standard deviation from the sample size, median, range and/or interquartile range. *BMC Med Res Methodol.* (2014) 14:135. 10.1186/1471-2288-14-135 25524443 PMC4383202

[26] Higgins JP Altman DG Gøtzsche PC Jüni P Moher D Oxman AD The Cochrane Collaboration’s tool for assessing risk of bias in randomised trials. *BMJ.* (2011) 343:d5928. 10.1136/bmj.d5928 22008217 PMC3196245

[27] Sterne JA Hernán MA Reeves BC Savović J Berkman ND Viswanathan M ROBINS-I: a tool for assessing risk of bias in non-randomised studies of interventions. *BMJ.* (2026) 355:i4919. 10.1136/bmj.i4919 27733354 PMC5062054

[28] Rücker G. Network meta-analysis, electrical networks and graph theory. *Res Synth Methods.* (2012) 3:312–24. 10.1002/jrsm.1058 26053424

[29] Rücker G Schwarzer G. Ranking treatments in frequentist network meta-analysis works without resampling methods. *BMC Med Res Methodol.* (2015) 15:58. 10.1186/s12874-015-0060-8 26227148 PMC4521472

[30] Chaimani A Salanti G. Using network meta-analysis to evaluate the existence of small-study effects in a network of interventions. *Res Synth Methods.* (2012) 3:161–76. 10.1002/jrsm.57 26062088

[31] Sedgwick P Marston L. How to read a funnel plot in a meta-analysis. *BMJ.* (2015) 351:h4718. 10.1136/bmj.h4718 26377337

[32] Papakonstantinou T Nikolakopoulou A Higgins JPT Egger M Salanti G. CINeMA: software for semiautomated assessment of the confidence in the results of network meta-analysis. *Campbell Syst Rev.* (2020) 16:e1080. 10.1002/cl2.1080 37131978 PMC8356302

[33] Nikolakopoulou A Higgins JPT Papakonstantinou T Chaimani A Del Giovane C Egger M CINeMA: an approach for assessing confidence in the results of a network meta-analysis. *PLoS Med.* (2020) 17:e1003082. 10.1371/journal.pmed.1003082 32243458 PMC7122720

[34] Watson A Zhang Y Beattie S Page RS. Prospective randomized controlled trial comparing dynamic hip screw and screw fixation for undisplaced subcapital hip fractures. *ANZ J Surg.* (2013) 83:679–83. 10.1111/j.1445-2197.2012.06256.x 22998439

[35] Siavashi B Aalirezaei A Moosavi M Golbakhsh MR Savadkoohi D Zehtab MJ. A comparative study between multiple cannulated screws and dynamic hip screw for fixation of femoral neck fracture in adults. *Int Orthop.* (2015) 39:2069–71. 10.1007/s00264-015-2881-9 26152248

[36] Gupta M Arya RK Kumar S Jain VK Sinha S Naik AK. Comparative study of multiple cancellous screws versus sliding hip screws in femoral neck fractures of young adults. *Chin J Traumatol.* (2016) 19:209–12. 10.1016/j.cjtee.2015.11.021 27578376 PMC4992136

[37] Hong H Sha M Chen Z Li Y Kang L. Comparison of dynamic hip screw with anti-rotation screw and femoral neck system internal fixation for the treatment of garden II-IV type femoral neck fractures. *Technol Health Care.* (2024) 32:4009–17. 10.3233/THC-231547 39031399 PMC11612970

[38] Nauth A Creek AT Zellar A Lawendy AR Dowrick A Gupta A Fracture fixation in the operative management of hip fractures (FAITH): an international, multicentre, randomised controlled trial. *Lancet.* (2017) 389:1519–27. 10.1016/S0140-6736(17)30066-1 28262269 PMC5597430

[39] Lee YS Chen SH Tsuang YH Huang HL Lo TY Huang CR. Internal fixation of undisplaced femoral neck fractures in the elderly: a retrospective comparison of fixation methods. *J Trauma.* (2008) 64:155–62. 10.1097/TA.0b013e31802c821c 18188115

[40] Jettoo P James P. Dynamic hip screw fixation versus multiple screw fixation for intracapsular hip fracture. *J Orthop Surg.* (2016) 24:146–9. 10.1177/1602400204 27574251

[41] Chen C Yu L Tang X Liu MZ Sun LZ Liu C Dynamic hip system blade versus cannulated compression screw for the treatment of femoral neck fractures: a retrospective study. *Acta Orthop Traumatol Turc.* (2017) 51:381–7. 10.1016/j.aott.2017.07.006 28844681 PMC6197598

[42] Şahin A Agar A Gülabi D Ertürk C. Comparison of dynamic hip screw and antirotation screw with cannulated screw in the treatment of transcervical collum femoris fractures. *Jt Dis Relat Surg.* (2020) 31:320–7. 10.5606/ehc.2020.73416 32584732 PMC7489184

[43] Hu H Cheng J Feng M Gao Z Wu J Lu S. Clinical outcome of femoral neck system versus cannulated compression screws for fixation of femoral neck fracture in younger patients. *J Orthop Surg Res.* (2021) 16:370. 10.1186/s13018-021-02517-z 34107990 PMC8188789

[44] Tang Y Zhang Z Wang L Xiong W Fang Q Wang G. Femoral neck system versus inverted cannulated cancellous screw for the treatment of femoral neck fractures in adults: a preliminary comparative study. *J Orthop Surg Res.* (2021) 16:504. 10.1186/s13018-021-02659-0 34399801 PMC8365927

[45] Zhou XQ Li ZQ Xu RJ She YS Zhang XX Chen GX Comparison of early clinical results for femoral neck system and cannulated screws in the treatment of unstable femoral neck fractures. *Orthop Surg.* (2021) 13:1802–9. 10.1111/os.13098 34351048 PMC8523763

[46] Zhang YZ Lin Y Li C Yue XJ Li GY Wang B A comparative analysis of femoral neck system and three cannulated screws fixation in the treatment of femoral neck fractures: a six-month follow-up. *Orthop Surg.* (2022) 14:686–93. 10.1111/os.13235 35179307 PMC9002068

[47] Abdallatif AG Sharma A Mahmood T Aslam N. Complications and outcomes of the internal fixation of non-displaced femoral neck fracture in old patients: a two-year follow-up. *Cureus.* (2023) 15:e41391. 10.7759/cureus.41391 37546038 PMC10401487

[48] Ge Z Xiong W Wang D Tang Y Fang Q Wang L Comparison of femoral neck system vs. dynamic hip system blade for the treatment of femoral neck fracture in young patients: a retrospective study. *Front Surg.* (2023) 10:1092786. 10.3389/fsurg.2023.1092786 36816012 PMC9935827

[49] Kenmegne GR Zou C Fang Y He X Lin Y Yin Y. Femoral neck fractures in non-geriatric patients: femoral neck system versus cannulated cancellous screw. *BMC Musculoskelet Disord.* (2023) 24:70. 10.1186/s12891-023-06140-3 36703126 PMC9878738

[50] Niemann M Maleitzke T Jahn M Salmoukas K Braun KF Graef F Restoration of hip geometry after femoral neck fracture: a comparison of the Femoral Neck System (FNS) and the Dynamic Hip Screw (DHS). *Life.* (2023) 13:2073. 10.3390/life13102073 37895454 PMC10608621

[51] Xu X Fan J Zhou F Lv Y Tian Y Ji H Comparison of femoral neck system to multiple cancellous screws and dynamic hip screws in the treatment of femoral neck fractures. *Injury.* (2023) 54:S28–35. 10.1016/j.injury.2022.03.041 35367076

[52] Yan SG Cui Y Li D Liu F Hua X Schmidutz F. Femoral neck system versus three cannulated screws for fixation of femoral neck fractures in younger patients: a retrospective cohort study. *J Invest Surg.* (2023) 36:2266752. 10.1080/08941939.2023.2266752 37813399

[53] Bukhary HA Aljuaid FI Alhomayani KM Saati AA Aldosari AM Hammadi WA Treatment of non-displaced intracapsular femoral neck fractures with dynamic hip and cannulated screws resulting in avascular necrosis: a comparative study of complications. *Saudi Med J.* (2024) 45:54–9. 10.15537/smj.2024.45.1.20230684 38220227 PMC10807667

[54] Caldaria A Gambuti E Biagi N Spadoni E Saracco A Massari L Comparison of femoral neck system versus cannulated cancellous screws for the fixation of femoral neck fracture: a single-center retrospective cohort study. *Eur J Orthop Surg Traumatol.* (2024) 34:3207–13. 10.1007/s00590-024-04051-0 39085468 PMC11377608

[55] Chung H Kim Y Kook I Kwak JW Hwang KT. Comparative short-term outcomes of Femoral Neck System (FNS) and cannulated screw fixation in patients with femoral neck fractures: a multicenter study. *Clin Orthop Surg.* (2024) 16:184–93. 10.4055/cios23190 38562623 PMC10973613

[56] Zheng S Lin D Chen P Lin C Chen B Zheng K Comparison of femoral neck shortening after femoral neck system and cannulated cancellous screw fixation for displaced femoral neck fractures in young adults. *Injury.* (2024) 55:111564. 10.1016/j.injury.2024.111564 38640596

[57] Harris WH. Traumatic arthritis of the hip after dislocation and acetabular fractures: treatment by mold arthroplasty. An end-result study using a new method of result evaluation. *J Bone Joint Surg Am.* (1969) 51:737–55. 5783851

[58] Zlowodzki M Brink O Switzer J Wingerter S Woodall J Petrisor BA The effect of shortening and varus collapse of the femoral neck on function after fixation of intracapsular fracture of the hip: a multi-centre cohort study. *J Bone Joint Surg Br.* (2008) 90:1487–94. 10.1302/0301-620X.90B11.20582 18978271

[59] Patel S Kumar V Baburaj V Dhillon MS. The use of the femoral neck system (FNS) leads to better outcomes in the surgical management of femoral neck fractures in adults compared to fixation with cannulated screws: a systematic review and meta-analysis. *Eur J Orthop Surg Traumatol.* (2023) 33:2101–9. 10.1007/s00590-022-03407-8 36201031

[60] Zielinski SM Keijsers NL Praet SF Heetveld MJ Bhandari M Wilssens JP Femoral neck shortening after internal fixation of a femoral neck fracture. *Orthopedics.* (2013) 36:e849–58. 10.3928/01477447-20130624-13 23823040

[61] Chen X Zhang J Wang X Ren J Liu Z. Incidence of and factors influencing femoral neck shortening in elderly patients after fracture fixation with multiple cancellous screws. *Med Sci Monit.* (2017) 23:1456–63. 10.12659/msm.899476 28343233 PMC5380194

[62] Yuan KX Yang F Fu K Zhu DY Jiang CY Jin DX Internal fixation using fully threaded cannulated compression screws for fresh femoral neck fractures in adults. *J Orthop Surg Res.* (2022) 17:108. 10.1186/s13018-022-03005-8 35184732 PMC8859893

[63] Mitsutake R Tanino H Sato G Ito H. Internal fixation versus total hip arthroplasty for displaced femoral neck fractures in patients aged 60 to 80 years: patient-reported outcomes and complications. *PLoS One.* (2025) 20:e0323106. 10.1371/journal.pone.0323106 40338864 PMC12061127

[64] Chen Y Kuan FC Hong CK Su WR Li HH Liu GY Evaluating barriers to achieving the minimal important change in older patients with hip fractures after post-acute care. *Geriatr Orthop Surg Rehabil.* (2025) 16:21514593251403425. 10.1177/21514593251403425 41312214 PMC12647561

[65] Ramadanov N Voss M Hable R Prill R Hakam HT Salzmann M Patient-related predictors for the functional outcome of SuperPATH hemiarthroplasty versus conventional approach hemiarthroplasty: a systematic review and meta-regression analysis of randomized controlled trials. *Orthop Surg.* (2024) 16:791–801. 10.1111/os.14006 38298174 PMC10984811

[66] Zhao F Guo L Wang X Zhang Y. Analysis on risk factors for neck shortening after internal fixation for Pauwels II femoral neck fracture in young patients. *Eur J Med Res.* (2021) 26:59. 10.1186/s40001-021-00531-9 34167592 PMC8223273

[67] Felton J Slobogean GP Jackson SS Della Rocca GJ Liew S Haverlag R Femoral neck shortening after hip fracture fixation is associated with inferior hip function: results from the FAITH trial. *J Orthop Trauma.* (2019) 33:487–96. 10.1097/BOT.0000000000001551 31464855

[68] Polat A Misir A Buyukkuscu MO Basilgan S Basar H. Factors associated with femoral neck shortening after closed or open reduction and screw fixation. *Indian J Orthop.* (2021) 56:303–11. 10.1007/s43465-021-00484-5 35140862 PMC8789974

[69] Sung YB Jung EY Kim KI Kim SY. Risk factors for neck shortening in patients with valgus impacted femoral neck fractures treated with three parallel screws: is bone density an affecting factor? *Hip Pelvis.* (2017) 29:277–85. 10.5371/hp.2017.29.4.277 29250503 PMC5729171

[70] Ramadanov N Toma I Herkner H Klein R Behringer W Matthes G. Factors that influence the complications and outcomes of femoral neck fractures treated by cannulated screw fixation. *Sci Rep.* (2020) 10:758. 10.1038/s41598-020-57696-2 31959840 PMC6971299

[71] Voeten S Deunk J Vermeulen J De Lange-De Klerk E van den Brand H Zuidema W. The addition of an anti-rotation screw to the dynamic hip screw. *Acta Orthop Belg.* (2020) 86:233–8. 33418612

[72] Memon K Siddiqui AM Khan ZA Zahoor A. Dynamic hip screw fixation vs. proximal femur nail for unstable per-trochanteric fractures: a comparative analysis of outcomes and complications. *J Ayub Med Coll Abbottabad.* (2021) 33:34–8. 33774951

[73] Chang JZ Xiao YP Li L Bei MJ. The efficacy of dynamic compression locking system vs. dynamic hip screw in the treatment of femoral neck fractures: a comparative study. *BMC Musculoskelet Disord.* (2022) 23:661. 10.1186/s12891-022-05631-z 35820870 PMC9275283

[74] Moon JK Lee JI Hwang KT Yang JH Park YS Park KC. Biomechanical comparison of the femoral neck system and the dynamic hip screw in basicervical femoral neck fractures. *Sci Rep.* (2022) 12:7915. 10.1038/s41598-022-11914-1 35551221 PMC9098555

[75] Teng Y Zhang Y Guo C. Finite element analysis of femoral neck system in the treatment of Pauwels type III femoral neck fracture. *Medicine.* (2022) 101:e29450. 10.1097/MD.0000000000029450 35839002 PMC11132412

[76] Zhang X Yang B Feng L Xu X Wang C Lee YW Augmenting osteoporotic bone regeneration through a hydrogel-based rejuvenating microenvironment. *Bioact Mater.* (2024) 41:440–54. 10.1016/j.bioactmat.2024.07.036 39188381 PMC11347042

[77] Cheung WH Miclau T Chow SK Yang FF Alt V. Fracture healing in osteoporotic bone. *Injury.* (2016) 47:S21–6. 10.1016/S0020-1383(16)47004-X 27338222

[78] Osterhoff G Morgan EF Shefelbine SJ Karim L McNamara LM Augat P. Bone mechanical properties and changes with osteoporosis. *Injury.* (2016) 47:S11–20. 10.1016/S0020-1383(16)47003-8 27338221 PMC4955555

[79] von Rüden C Augat P. Failure of fracture fixation in osteoporotic bone. *Injury.* (2016) 47:S3–10. 10.1016/S0020-1383(16)47002-6 27338224

[80] Anderson KD Ko FC Virdi AS Sumner DR Ross RD. Biomechanics of implant fixation in osteoporotic bone. *Curr Osteoporos Rep.* (2020) 18:577–86. 10.1007/s11914-020-00614-2 32734511 PMC7541774

[81] Fisher JC Gerzina C Rush K Caroom C. Subtrochanteric fracture after femoral neck system of femoral neck fractures: a report of four cases. *BMC Musculoskelet Disord.* (2023) 24:749. 10.1186/s12891-023-06872-2 37737167 PMC10514930

[82] Hsu MR Shu HT Luksameearunothai K Margalit A Yu AT Hasenboehler EA Is there an increased risk for subtrochanteric stress fracture with the Femoral Neck System versus multiple cannulated screws fixation? *J. Orthop.* (2022) 30:127–33. 10.1016/j.jor.2022.02.016 35280450 PMC8907549

[83] Fan X Zhou Y Dai S Lao K Zhang Q Yu T. Bio-mechanical effects of femoral neck system versus cannulated screws on treating young patients with Pauwels type III femoral neck fractures: a finite element analysis. *BMC Musculoskelet Disord.* (2024) 25:83. 10.1186/s12891-023-07110-5 38245678 PMC10799488

[84] Jung CH Cha Y Chung JY Park CH Kim TY Yoo JI Trajectory of bolt and length of plate in femoral neck system determine the stability of femur neck fracture and risk of subsequent subtrochanteric fracture: a finite element analysis. *BMC Musculoskelet Disord.* (2023) 24:465. 10.1186/s12891-023-06579-4 37280558 PMC10245606

[85] Li N Cheng KY Fan J Li Y Yang M Zhu S Evaluating three internal fixation techniques for Pauwels III femoral neck fractures via finite element analysis. *Sci Rep.* (2024) 14:15519. 10.1038/s41598-024-66638-1 38969693 PMC11226618

[86] Stoffel K Michelitsch C Arora R Babst R Candrian C Eickhoff A Clinical performance of the Femoral Neck System within 1 year in 125 patients with acute femoral neck fractures, a prospective observational case series. *Arch Orthop Trauma Surg.* (2023) 143:4155–64. 10.1007/s00402-022-04686-w 36460761 PMC10293436

[87] Nan C Liu Y Zhang D Qin Y Yu H Liu Y Mechanical analysis of modified femoral neck system in the treatment of osteoporotic femoral neck fractures. *BMC Musculoskelet Disord.* (2024) 25:789. 10.1186/s12891-024-07907-y 39367368 PMC11452960

