# Supplementary material for: Comparative efficacy and safety of three internal fixation strategies for femoral neck fractures: a network meta-analysis
Source: Front Med (Lausanne). 2026 Mar 2;13:1782357. doi: 10.3389/fmed.2026.1782357 (PMC12989485; doi:10.3389/fmed.2026.1782357)
Supplement: Supplementary file 1 [file Image_1.pdf]

## Supplementary information 1

# Comparison of the Effects of Three Internal Fixation Methods for Femoral Neck Fractures: A Network Meta-analysis

## Content


Table S1: Reason for exclusion at the full-text screening stage ........ 3
Fig. S1: Risk of bias assessment for randomized control studies ........ 6
Table S2: Summary of Risk of Bias Assessment for Included Cohort Studies ........ 7
**Pairwise Meta-analysis Forest Plots** ........ 9
Fig. S2: Harris Hip Score ........ 9
Fig. S4: Femoral head necrosis ........ 10
Fig. S6: Femoral neck shortening ........ 10
Fig. S8: Implant failure/cut-out ........ 11
Fig. S10: Fracture nonunion/delayed union ........ 11
Fig. S12: Intraoperative blood loss ........ 12
Fig. S14: Operative time ........ 12
Fig. S16: Fracture healing time ........ 13
**Network Meta-analysis Forest Plots** ........ 14
Fig. S3: Harris Hip Score ........ 14
Fig. S5: Femoral head necrosis ........ 14
Fig. S7: Femoral neck shortening ........ 15
Fig. S9: Implant failure/cut-out ........ 15
Fig. S11: Fracture nonunion/delayed union ........ 16
Fig. S13: Intraoperative blood loss ........ 16
Fig. S15: Operative time ........ 17
Fig. S17: Fracture healing time ........ 17
**Fig. S18: Funnel plots for primary and secondary outcomes** ........ 18
**Node-Splitting Analysis** ........ 22
Fig. S19: Harris Hip Score ........ 22
Fig. S20: Femoral head necrosis ........ 23
Fig. S21: Femoral neck shortening ........ 24
Fig. S22: Implant failure/cut-out ........ 25
Fig. S23: Fracture nonunion/delayed union ........ 26
Fig. S24: Intraoperative blood loss ........ 27
Fig. S25: Operative time ........ 28
Fig. S26: Fracture healing time ........ 29
**Sensitivity and exploratory analyses** ........ 30
Fig. S27: Harris Hip Score ........ 30
Fig. S28: Femoral head necrosis ........ 31
Fig. S29: Implant failure/cut-out ........ 32
Fig. S30: Fracture nonunion/delayed union ........ 33
Fig. S31: Intraoperative blood loss ........ 34
Fig. S32: Operative time ........ 35
Table S3: Results of Subgroup Analyses ........ 36
Table S4: The results of meta-regression based on age ........ 37
Table S5: CINeMA Confidence Ratings for Primary and Secondary outcomes ........ 38

## Search strategy

### Pubmed

#1 "femoral neck fractures"[MeSH Terms]

#2 "femoral neck fracture"[Title/Abstract] OR "femur neck fracture"[Title/Abstract] OR "intracapsular hip fracture"[Title/Abstract]

#3 #1 AND #2

#4 "internal fixators"[MeSH Terms] OR "bone screws"[MeSH Terms] OR "fracture fixation, internal"[MeSH Terms]

#5 "femoral neck system"[Title/Abstract] OR "FNS"[Title/Abstract]

#6 "cannulated compression screw"[Title/Abstract] OR "CCS"[Title/Abstract] OR "cannulated screw"[Title/Abstract] OR "multiple screw fixation"[Title/Abstract]

#7 "dynamic hip screw"[Title/Abstract] OR "DHS"[Title/Abstract] OR "SHS"[Title/Abstract] OR "sliding hip screw"[Title/Abstract]

#8 #4 OR #5 OR #6 OR #7

#9 #3 AND #8

### Embase

#1 'femur neck fracture'/exp

#2'femoral neck fracture':ti,ab OR 'femur neck fracture':ti,ab OR 'intracapsular hip fracture':ti,ab

#3 #1 AND #2

#4 'internal fixator'/exp OR 'internal fixator' OR 'bone screw'/exp OR 'bone screw' OR 'osteosynthesis'/exp OR 'osteosynthesis'

#5 'femoral neck system':ti,ab OR 'fns':ti,ab

#6 'cannulated compression screw':ti,ab OR 'ccs':ti,ab OR 'cannulated screw':ti,ab OR 'multiple screw fixation':ti,ab

#7 'dynamic hip screw':ti,ab OR 'dhs':ti,ab OR 'sliding hip screw':ti,ab OR 'shs':ti,ab

#8 #4 OR #5 OR #6 OR #7

#9 #3 AND #8

### Cochrane Library

#1 MeSH descriptor: [Femoral Neck Fractures] explode all trees

#2 (femoral neck fracture OR femur neck fracture OR intracapsular hip fracture):ti,ab,kw

#3 #1 OR #2

#4 MeSH descriptor: [Internal Fixators] explode all trees

#5 MeSH descriptor: [Bone Screws] explode all trees

#6 (femoral neck system OR FNS):ti,ab,kw

#7 (cannulated compression screw OR CCS OR cannulated screw OR multiple screw fixation):ti,ab,kw

#8 (dynamic hip screw OR DHS OR sliding hip screw OR SHS):ti,ab,kw

#9 #4 OR #5 OR #6 OR #7 OR #8

#10 #3 AND #9

**Table S1: Reason for exclusion at the full-text screening stage**

| Study | Title | Reason for exclusion |
|---|---|---|
| Min BW et al. 1999 | Femoral Neck Fracture Fixation (Comparison of Dynamic Hip Screw and Cannulated Screw Fixation) | No full text |
| Wu CC et al. 2003 | Minimally displaced intra-capsular femoral neck fractures in the elderly--comparison of multiple threaded pins and sliding compression screws surgical techniques | Wrong intervention |
| Heetveld MJ et al. 2007 | Rating of internal fixation and clinical outcome in displaced femoral neck fractures: a prospective multicenter study | No interested outcomes |
| Ntr 2010 | RSA study on femoral neck fractures: DHS versus three cannulated hip screws | No interested outcomes |
| Chen Z et al. 2011 | [Efficacy comparison between dynamic hip screw combined with anti-rotation screw and cannulated screw in treating femoral neck fractures] | Non-English |
| Bhandari M 2014 | Fixation using alternative implants for the treatment of hip fractures (FAITH): design and rationale for a multi-centre randomized trial comparing sliding hip screws and cancellous screws on revision surgery rates and quality of life in the treatment of femoral neck fractures | Wrong study design |
| Hou WR et al. 2015 | [Comparison among three cannulated screws and dynamic hip screw combined with antirotation screw for comminuted fractures of femoral neck] | Non-English |
| Stengel D 2017 | [Cancellous screws versus dynamic hip screw fixation of femural neck fractures : The Fixation using Alternative Implants for the Treatment of Hip fractures Study (FAITH)] | Wrong study design |
| Bisaccia M et al. 2018 | Dhs plus anti-rotational screw vs cannulated screws for femoral neck fractures: an analysis of clinical outcome and incidence regarding avn | No interested outcomes |
| Slobogean GP et al. 2019 | Fixation using alternative implants for the treatment of hip fractures (FAITH-2): design and rationale for a pilot multi-centre 2 × 2 factorial randomized controlled trial in young femoral neck fracture patients | Wrong study design |
| Nct 2020 | A Prospective Multicenter Cohort Study About Internal Fixation Using FNS Versus MCS for Femoral Neck Fracture | No interested outcomes |
| Shokri A et al. 2020 | Comparing therapeutic outcomes of dynamic hip screw and multiple cannulated screws as internal fixation in femoral neck fractures | Non-English |
| Slobogean GP et al. 2020 | Fixation Using Alternative Implants for the Treatment of Hip Fractures (FAITH-2): the Clinical Outcomes of a Multicenter 2 × 2 Factorial Randomized Controlled | Wrong intervention |

| | | |
|---|---|---|
| | Pilot Trial in Young Femoral Neck Fracture Patients | |
| Ctri 2021 | Prospective Randomized Controlled Trial to compare Femoral neck shortening after two different treatment modalities | No full text |
| Yan C et al. 2021 | Comparison of effectiveness of femoral neck system and cannulate compression screw in treatment of femoral neck fracture in young and middle-aged patients | Non-English |
| Yang J et al. 2021 | Comparison of femoral neck system and inverted triangle cannulated screws fixations in treatment of Pauwels typle Ⅲ femoral neck fractures | Non-English |
| Lu Y et al. 2022 | Is Femoral Neck System a Valid Alternative for the Treatment of Displaced Femoral Neck Fractures in Adolescents? A Comparative Study of Femoral Neck System versus Cannulated Compression Screw | Improper patients |
| Nibe Y et al. 2022 | A comparison between the femoral neck system and other implants for elderly patients with femoral neck fracture: A preliminary report of a newly developed implant | Wrong intervention |
| Niemann M et al. 2022 | Comparing Perioperative Outcome Measures of the Dynamic Hip Screw and the Femoral Neck System | No interested outcomes |
| Ullah SH et al. 2022 | Comparison of Outcome of DHS (Dynamic Hip Screw) Vs Cannulated Screws in Managing Fracture of Neck of Femur | No interested outcomes |
| Viberg B et al. 2022 | Reoperation for sliding hip screws vs cannulated cancellous screws in femoral neck fractures: A study from the Danish Fracture Database Collaborators | No interested outcomes |
| ChiCtr 2023 | Comparison of clinical results for femoral neck system and cannulated compression screws in the treatment of nondisplaced femoral neck fractures in elderly patients | No full text |
| Lin H et al. 2023 | Femoral Neck System vs. four cannulated screws in the treatment of Pauwels III femoral neck fracture | Wrong intervention |
| Schuetze K et al. 2023 | Is new always better: comparison of the femoral neck system and the dynamic hip screw in the treatment of femoral neck fractures | Insufficient follow-up duration |
| Tctr 2023 | Clinical outcomes of femoral neck fracture fixation: femoral neck system versus Cannulated screws, A randomized control trial | No full text |
| Tctr 2023 | Pauwels screw combined inverted triangle multiple screws vs. Dynamic hip screw for femoral neck fracture Pauwels classification II-III | No full text |
| Wang T et al. 2023 | Comparison of short-term follow-up results between femoral neck system and cannulated compressive screws in the treatment of femoral neck fracture in the elderly | Non-English |

| | | |
|---|---|---|
| Xiao H et al. 2023 | Comparison of dynamic hip screw and anti-rotation screw internal fixation and femoral neck system internal fixation in the treatment of Garden II-IV femoral neck fracture | Non-English |
| Zhang F et al. 2023 | Short term follow-up of unstable femoral neck fractures in young adults treated with femoral neck system and cannulated compression screws | Non-English |
| Cai L et al. 2024 | Comparison of young femoral neck fractures treated by femoral neck system, multiple cancellous screws and dynamic hip screws: a retrospectively comparison study | Wrong intervention |
| Ctri 2024 | Femoral Neck System or Cannulated Cancellous Screws which is better for management of intracapsular neck femur fracture | No full text |
| Gupta S et al. 2024 | Difference of Neck Shortening in Femoral Neck Fracture between Femoral Neck System and Multiple Cannulated Cancellous Screws: single Center, Prospective Randomized Controlled Trial | No full text |
| Honkanen J et al. 2024 | Comparable results for the Femoral Neck System and three-screw fixation in femoral neck fracture treatment | No interested outcomes |
| Jiang T et al. 2024 | The comparison of femoral neck system and cancellous screws internal fixation for femoral neck fracture | No full text |
| Lalueza-Andreu P et al. 2024 | Surgical treatment of non-displaced subcapital hip fracture: Femoral Neck System vs. cannulated screws. Comparative study | Non-English |
| Rathod H et al. 2024 | A comparison and evaluation of dynamic hip screws versus multiple cannulated cancellous screws for treating femoral neck fractures in young Indian individuals | Improper patients |
| Wang Q et al. 2024 | [Short term follow-up of femoral neck dynamic cross screw system and threaded cannulated screw in the treatment of vertically unstable femoral neck fractures] | Non-English |
| Yeoh SC et al. 2024 | Femoral neck system versus multiple cannulated screws for the fixation of Pauwels classification type II femoral neck fractures in older female patients with low bone mass | Wrong intervention |
| Zhai Q et al. 2024 | Revision rate of displaced femoral neck fractures in non-elderly adults: a retrospective comparative study of the femoral neck system and cannulated screws | No interested outcomes |
| Zhang X et al. 2024 | Comparison of three different internal fixation methods in the treatment of femoral neck fracture | Improper patients |
| Gao Y et al. 2025 | Femoral neck system (FNS) versus 4 cannulated compression screws (CCSs) in the treatment of young patients with Pauwels type III femoral neck fracture: a retrospective comparative study | Wrong intervention |

**Fig. S1: Risk of bias assessment for randomized control studies**

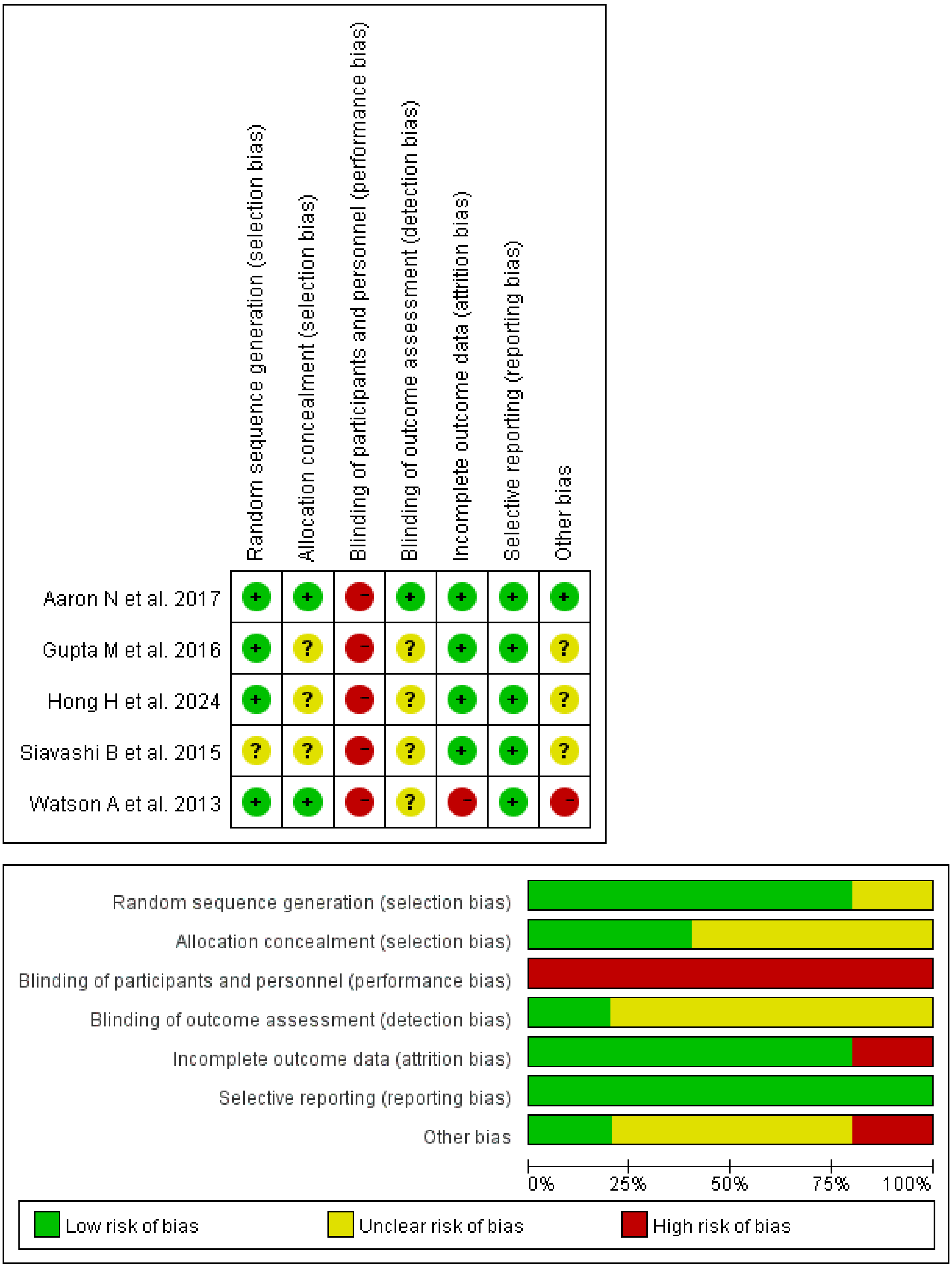

## Table S2: Summary of Risk of Bias Assessment for Included Cohort Studies Using the ROBINS-I Tool

**Table S2: Summary of Risk of Bias Assessment for Included Cohort Studies Using the ROBINS-I Tool**

| Study | Bias due to confounding | Bias in selection of participants | Bias in classification of interventions | Bias due to deviations from intended interventions | Bias due to missing data | Bias in measurement of outcomes | Bias in selection of the reported result | Overall risk of bias |
|---|---|---|---|---|---|---|---|---|
| Lee YS et al. 2008 | 🟡 | 🟢 | 🟢 | 🟡 | 🟡 | 🟡 | 🟡 | 🟡 |
| Jettoo P et al. 2016 | 🔴 | 🟢 | 🟡 | 🟢 | 🟢 | 🟡 | 🟡 | 🔴 |
| Chen C et al. 2017 | 🟡 | 🟢 | 🟢 | 🟢 | 🟡 | 🟡 | 🟡 | 🟡 |
| Sahin A et al. 2020 | 🟡 | 🟢 | 🟢 | 🟢 | 🟡 | 🟢 | 🟡 | 🟡 |
| Hu H et al. 2021 | 🟡 | 🟢 | 🟢 | 🟢 | 🟢 | 🟡 | 🟡 | 🟡 |
| Tang Y et al. 2021 | 🟡 | 🟢 | 🟢 | 🟢 | 🟢 | 🟡 | 🟡 | 🟡 |
| Zhou XQ et al. 2021 | 🟡 | 🟢 | 🟢 | 🟢 | 🟢 | 🟡 | 🟡 | 🟡 |
| Zhang YZ et al. 2022 | 🟡 | 🟢 | 🟢 | 🟢 | 🟢 | 🟡 | 🟡 | 🟡 |
| Abdallatif AG et al. 2023 | 🟡 | 🟢 | 🟢 | 🟢 | 🟡 | 🟡 | 🟡 | 🟡 |
| Ge Z et al. 2023 | 🟡 | 🟡 | 🟢 | 🟢 | 🟡 | 🟢 | 🟡 | 🟡 |

| | | | | | | | | |
|---|---|---|---|---|---|---|---|---|
| Kenmegne GR et al. 2023 | 🟡 | 🟡 | 🟢 | 🟢 | 🟢 | 🟡 | 🟡 | 🟡 |
| Niemann M et al. 2023 | 🟡 | 🟡 | 🟢 | 🟢 | 🟡 | 🟡 | 🟡 | 🟡 |
| Xu X et al. 2023 | 🟡 | 🟡 | 🟢 | 🟢 | 🟢 | 🟡 | 🟡 | 🟡 |
| Yan SG et al. 2023 | 🟡 | 🟡 | 🟢 | 🟢 | 🟡 | 🟡 | 🟡 | 🟡 |
| Bukhary HA et al. 2024 | 🟡 | 🟡 | 🟢 | 🟡 | 🟡 | 🟢 | 🟡 | 🟡 |
| Caldaria A et al. 2024 | 🟡 | 🟢 | 🟢 | 🟡 | 🟢 | 🟡 | 🟡 | 🟡 |
| Chung H et al. 2024 | 🟡 | 🟡 | 🟢 | 🟢 | 🟡 | 🟢 | 🟡 | 🟡 |
| Zheng S et al. 2024 | 🟡 | 🟢 | 🟢 | 🟡 | 🟡 | 🟡 | 🟢 | 🟡 |

Note: 🟢 = Low risk of bias; 🟡 = Moderate risk of bias; 🔴 = Serious risk of bias.

## Pairwise Meta-analysis Forest Plots

This section presents the direct, head-to-head comparisons between two intervention groups for each outcome. Results are displayed as traditional forest plots.

### Harris Hip Score

Fig. S2: Harris Hip Score pairwise forest plot

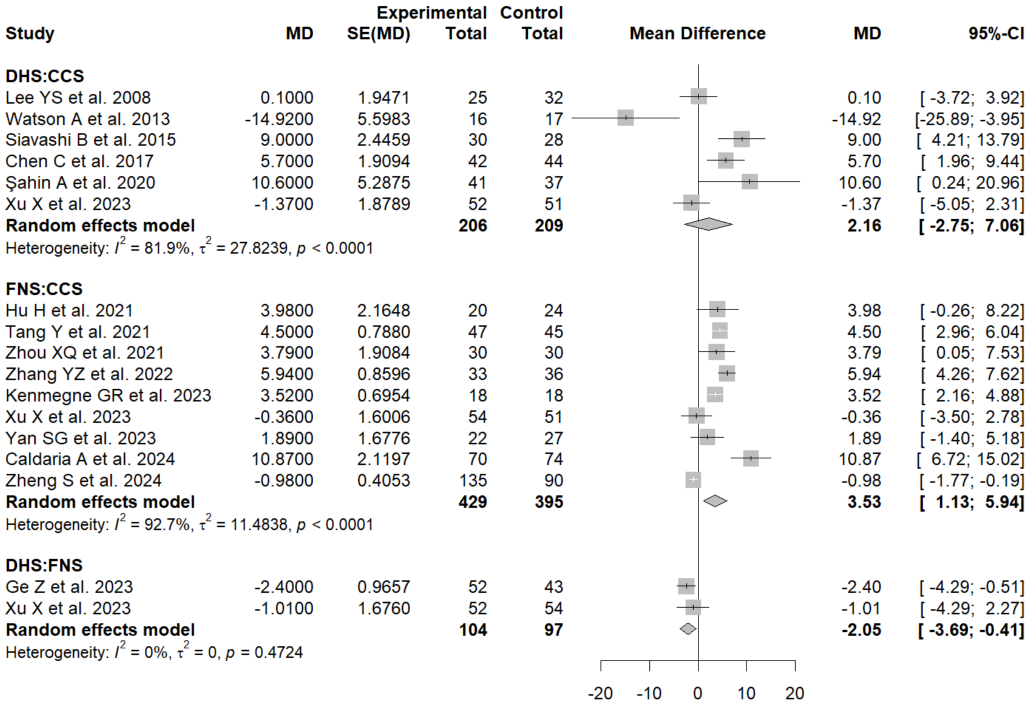

## Femoral head necrosis

Fig. S4: Femoral head necrosis pairwise forest plot

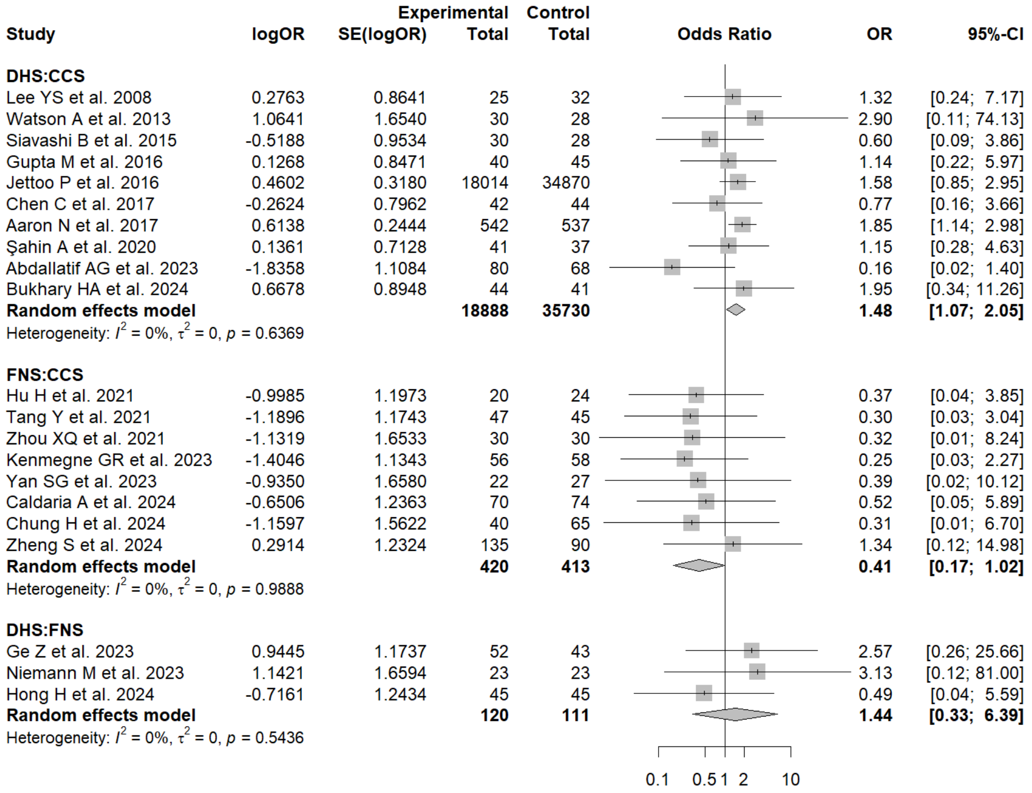


## Femoral neck shortening

Fig. S6: Femoral neck shortening pairwise forest plot

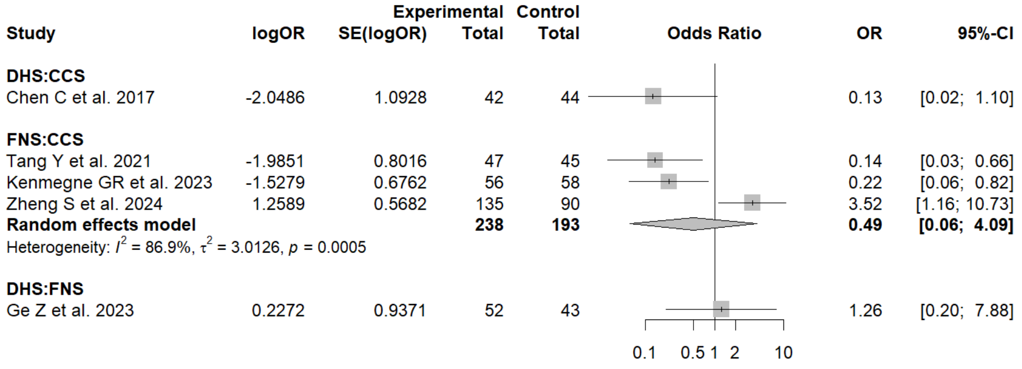

## Implant failure/cut-out

Fig. S8: Implant failure/cut-out pairwise forest plot

| Study | logOR | SE(logOR) | Experimental Total | Control Total | OR | 95%-CI |
|---|---|---|---|---|---|---|
| **DHS:CCS** | | | | | | |
| Lee YS et al. 2008 | -1.8002 | 1.5358 | 25 | 32 | 0.17 | [0.01; 3.35] |
| Watson A et al. 2013 | -0.0715 | 1.4393 | 30 | 28 | 0.93 | [0.06; 15.63] |
| Siavashi B et al. 2015 | -2.6586 | 1.5024 | 30 | 28 | 0.07 | [0.00; 1.33] |
| Gupta M et al. 2016 | 0.3814 | 0.7092 | 40 | 45 | 1.46 | [0.36; 5.88] |
| Chen C et al. 2017 | -2.0149 | 0.7997 | 42 | 44 | 0.13 | [0.03; 0.64] |
| Aaron N et al. 2017 | -0.0851 | 0.2238 | 542 | 537 | 0.92 | [0.59; 1.42] |
| Şahin A et al. 2020 | -0.8267 | 1.2464 | 41 | 37 | 0.44 | [0.04; 5.03] |
| Xu X et al. 2023 | 1.0986 | 1.6448 | 52 | 51 | 3.00 | [0.12; 75.37] |
| **Random effects model** | | | **802** | **802** | **0.58** | **[0.27; 1.25]** |
| Heterogeneity: $I^2 = 34.5\%$, $\tau^2 = 0.3626$, $p = 0.1526$ | | | | | | |
| **FNS:CCS** | | | | | | |
| Hu H et al. 2021 | -2.6676 | 1.5022 | 20 | 24 | 0.07 | [0.00; 1.32] |
| Tang Y et al. 2021 | -0.6061 | 0.7623 | 47 | 45 | 0.55 | [0.12; 2.43] |
| Zhou XQ et al. 2021 | -2.0495 | 1.5346 | 30 | 30 | 0.13 | [0.01; 2.61] |
| Kenmegne GR et al. 2023 | -0.9350 | 0.8587 | 56 | 58 | 0.39 | [0.07; 2.11] |
| Xu X et al. 2023 | 1.9459 | 1.5246 | 54 | 51 | 7.00 | [0.35; 138.96] |
| Yan SG et al. 2023 | -2.1795 | 1.1069 | 22 | 27 | 0.11 | [0.01; 0.99] |
| Caldaria A et al. 2024 | -1.3719 | 1.1308 | 70 | 74 | 0.25 | [0.03; 2.33] |
| Chung H et al. 2024 | 0.2877 | 0.7032 | 40 | 65 | 1.33 | [0.34; 5.29] |
| Zheng S et al. 2024 | -0.4092 | 1.4208 | 135 | 90 | 0.66 | [0.04; 10.76] |
| **Random effects model** | | | **474** | **464** | **0.47** | **[0.22; 1.01]** |
| Heterogeneity: $I^2 = 17.9\%$, $\tau^2 = 0.2424$, $p = 0.2834$ | | | | | | |
| **DHS:FNS** | | | | | | |
| Ge Z et al. 2023 | 0.5188 | 1.2425 | 52 | 43 | 1.68 | [0.15; 19.18] |
| Xu X et al. 2023 | -0.8473 | 0.9956 | 52 | 54 | 0.43 | [0.06; 3.02] |
| Hong H et al. 2024 | -1.1451 | 1.1747 | 45 | 45 | 0.32 | [0.03; 3.18] |
| **Random effects model** | | | **149** | **142** | **0.57** | **[0.16; 2.02]** |
| Heterogeneity: $I^2 = 0\%$, $\tau^2 = 0$, $p = 0.5813$ | | | | | | |

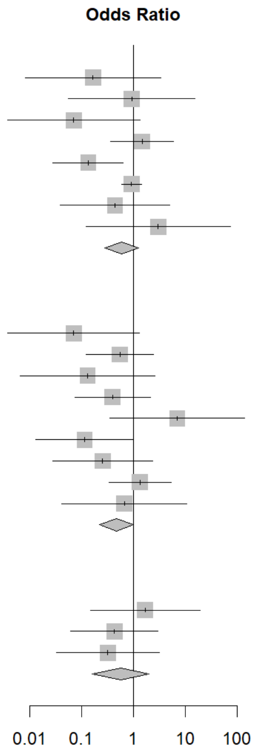


## Fracture nonunion/delayed union

Fig. S10: Fracture nonunion/delayed union pairwise forest plot

| Study | logOR | SE(logOR) | Experimental Total | Control Total | OR | 95%-CI |
|---|---|---|---|---|---|---|
| **DHS:CCS** | | | | | | |
| Lee YS et al. 2008 | -0.8873 | 1.6546 | 25 | 32 | 0.41 | [0.02; 10.54] |
| Watson A et al. 2013 | -1.7500 | 1.5718 | 30 | 28 | 0.17 | [0.01; 3.78] |
| Gupta M et al. 2016 | -0.4144 | 0.6169 | 40 | 45 | 0.66 | [0.20; 2.21] |
| Jettoo P et al. 2016 | -0.2929 | 0.0742 | 18014 | 34870 | 0.75 | [0.65; 0.86] |
| Chen C et al. 2017 | -1.6094 | 1.5643 | 42 | 44 | 0.20 | [0.01; 4.29] |
| Aaron N et al. 2017 | -0.0099 | 0.2541 | 542 | 537 | 0.99 | [0.60; 1.63] |
| Şahin A et al. 2020 | -0.6862 | 0.6223 | 41 | 37 | 0.50 | [0.15; 1.70] |
| Abdallatif AG et al. 2023 | -1.8358 | 1.1084 | 80 | 68 | 0.16 | [0.02; 1.40] |
| Xu X et al. 2023 | -0.5504 | 1.0518 | 52 | 51 | 0.58 | [0.07; 4.53] |
| Bukhary HA et al. 2024 | -0.0253 | 1.0250 | 42 | 41 | 0.98 | [0.13; 7.27] |
| **Random effects model** | | | **18908** | **35753** | **0.75** | **[0.65; 0.86]** |
| Heterogeneity: $I^2 = 0\%$, $\tau^2 = 0$, $p = 0.7943$ | | | | | | |
| **FNS:CCS** | | | | | | |
| Hu H et al. 2021 | -0.2513 | 0.9677 | 20 | 24 | 0.78 | [0.12; 5.18] |
| Tang Y et al. 2021 | -0.7862 | 0.8925 | 47 | 45 | 0.46 | [0.08; 2.62] |
| Zhou XQ et al. 2021 | -1.1701 | 1.1853 | 30 | 30 | 0.31 | [0.03; 3.17] |
| Kenmegne GR et al. 2023 | -0.9350 | 0.8587 | 56 | 58 | 0.39 | [0.07; 2.11] |
| Xu X et al. 2023 | -1.7057 | 1.5616 | 54 | 51 | 0.18 | [0.01; 3.88] |
| Yan SG et al. 2023 | -0.9350 | 1.6580 | 22 | 27 | 0.39 | [0.02; 10.12] |
| Chung H et al. 2024 | -1.7795 | 1.5044 | 40 | 65 | 0.17 | [0.01; 3.22] |
| Zheng S et al. 2024 | -0.4092 | 1.4208 | 135 | 90 | 0.66 | [0.04; 10.76] |
| **Random effects model** | | | **404** | **390** | **0.41** | **[0.19; 0.91]** |
| Heterogeneity: $I^2 = 0\%$, $\tau^2 = 0$, $p = 0.9899$ | | | | | | |
| **DHS:FNS** | | | | | | |
| Ge Z et al. 2023 | 0.2272 | 0.9371 | 52 | 43 | 1.26 | [0.20; 7.88] |
| Xu X et al. 2023 | 1.1552 | 1.6445 | 52 | 54 | 3.17 | [0.13; 79.71] |
| Hong H et al. 2024 | -0.7408 | 0.8931 | 45 | 45 | 0.48 | [0.08; 2.74] |
| **Random effects model** | | | **149** | **142** | **0.92** | **[0.28; 2.98]** |
| Heterogeneity: $I^2 = 0\%$, $\tau^2 = 0$, $p = 0.5437$ | | | | | | |

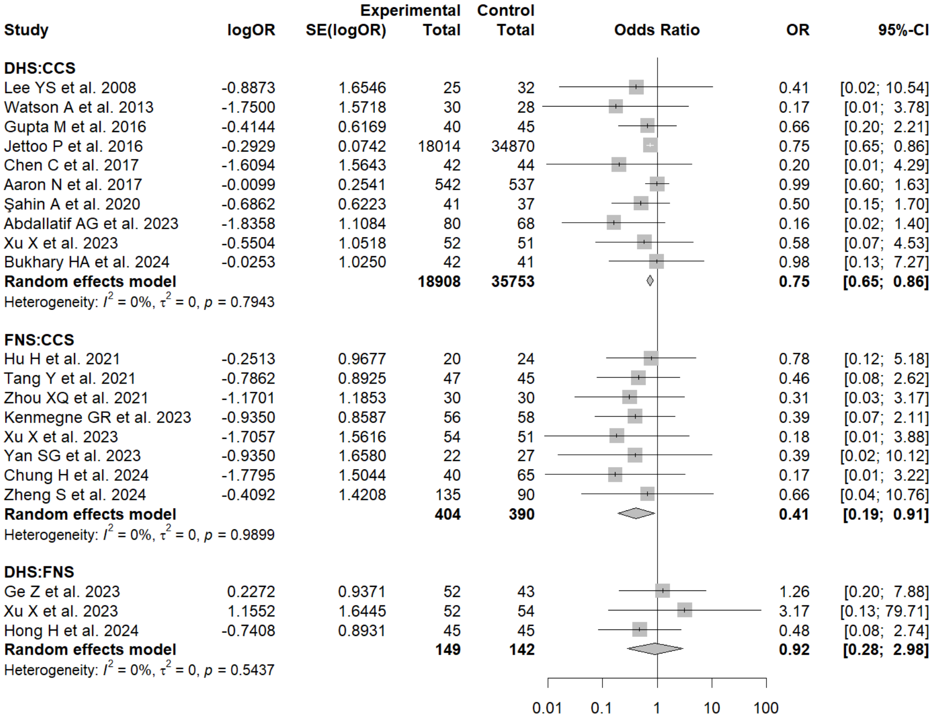

## Intraoperative blood loss

Fig. S12: Intraoperative blood loss pairwise forest plot

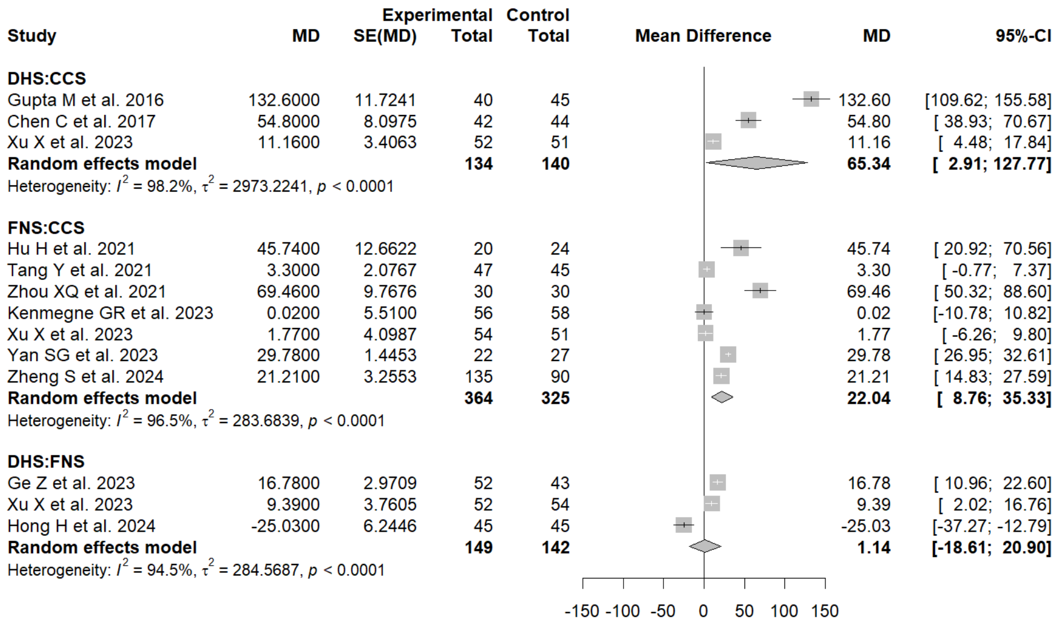


## Operative time

Fig. S14: Operative time pairwise forest plot

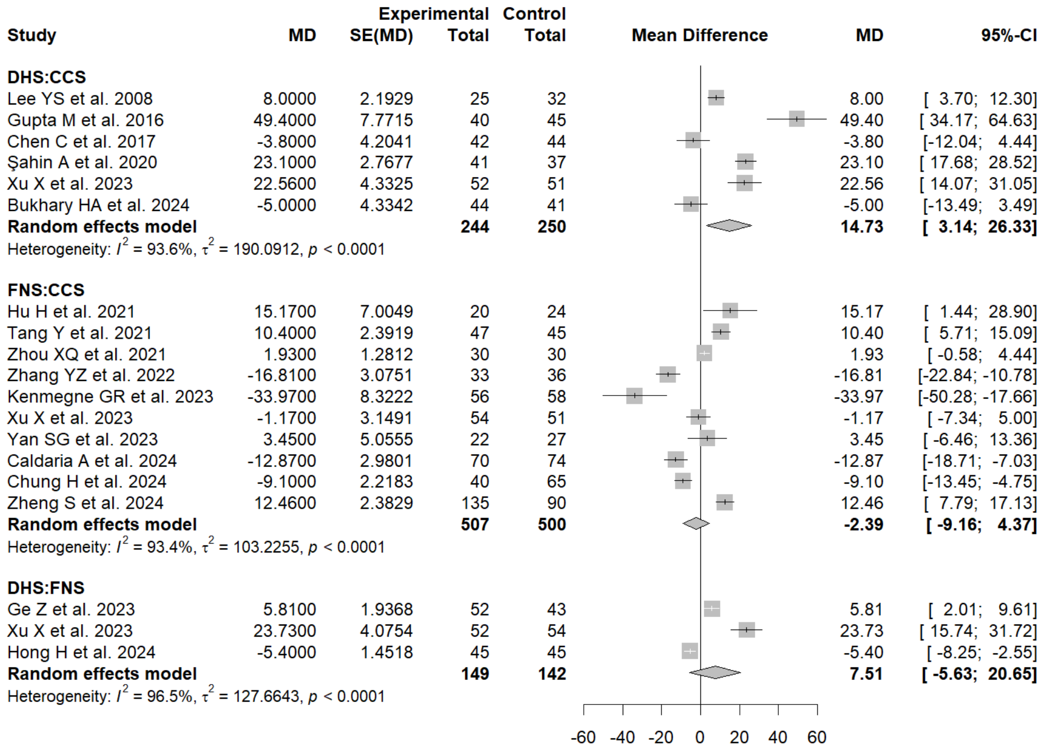

## Fracture healing time

Fig. S16: Fracture healing time pairwise forest plot

| Study | MD | SE(MD) | Experimental Total | Control Total | MD | 95%-CI |
|---|---|---|---|---|---|---|
| **DHS:CCS** | | | | | | |
| Lee YS et al. 2008 | -0.1400 | 0.1008 | 25 | 32 | -0.14 | [-0.34; 0.06] |
| **FNS:CCS** | | | | | | |
| Hu H et al. 2021 | -0.6100 | 0.2881 | 20 | 24 | -0.61 | [-1.17; -0.05] |
| Tang Y et al. 2021 | -1.0300 | 0.0774 | 47 | 45 | -1.03 | [-1.18; -0.88] |
| Kenmegne GR et al. 2023 | -2.2400 | 0.1480 | 56 | 58 | -2.24 | [-2.53; -1.95] |
| Yan SG et al. 2023 | -0.7000 | 0.1446 | 22 | 27 | -0.70 | [-0.98; -0.42] |
| Chung H et al. 2024 | -0.7000 | 0.2442 | 40 | 65 | -0.70 | [-1.18; -0.22] |
| **Random effects model** | | | **185** | **219** | **-1.07** | **[-1.65; -0.50]** |
| Heterogeneity: $I^2 = 94.5\%$, $\tau^2 = 0.3921$, $p < 0.0001$ | | | | | | |
| **DHS:FNS** | | | | | | |
| Hong H et al. 2024 | 0.1700 | 0.1888 | 45 | 45 | 0.17 | [-0.20; 0.54] |

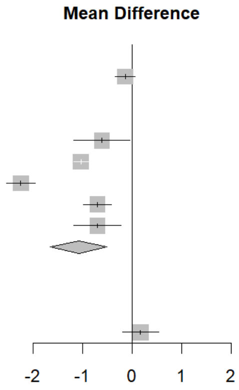

## Network Meta-analysis Forest Plots.

This section presents the league tables and forest plots from the network meta-analysis, showing the comparative effects of all three interventions.

### Harris Hip Score

Fig. S3: Forest plots for Harris Hip Score from the network meta-analysis.

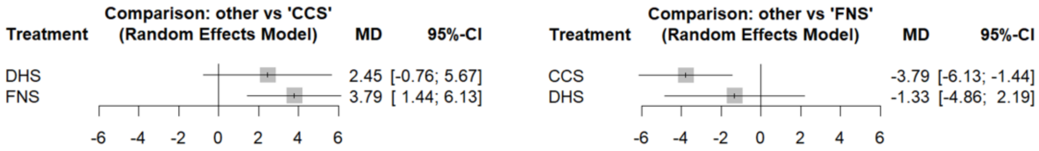


Treatment estimate:

| | MD | 95%-CI | z | p-value |
|---|---|---|---|---|
| DHS vs FNS | -1.3339 | [-4.8598; 2.1920] | -0.74 | 0.4584 |
| DHS vs CCS | 2.4514 | [-0.7643; 5.6671] | 1.49 | 0.1351 |
| FNS vs CCS | 3.7853 | [ 1.4367; 6.1339] | 3.16 | 0.0016 |

Quantifying heterogeneity / inconsistency:
tau^2 = 12.1455; tau = 3.4850; I^2 = 90.1% [85.4%; 93.3%]

Tests of heterogeneity (within designs) and inconsistency (between designs):

| | Q | d.f. | p-value |
|---|---|---|---|
| Total | 141.76 | 14 | < 0.0001 |
| Within designs | 130.45 | 11 | < 0.0001 |
| Between designs | 11.31 | 3 | 0.0102 |

Details of network meta-analysis methods:
- Frequentist graph-theoretical approach
- DerSimonian-Laird estimator for tau^2
- Calculation of I^2 based on Q

### Femoral head necrosis

Fig. S5: Forest plots for femoral head necrosis from the network meta-analysis.

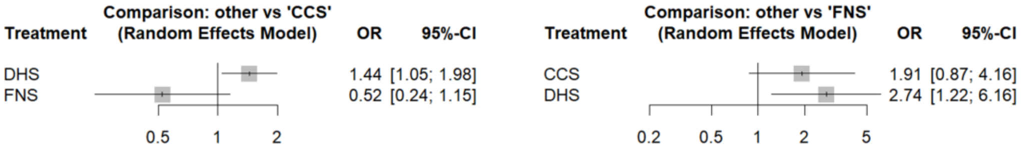


Treatment estimate:

| | MD | 95%-CI | z | p-value |
|---|---|---|---|---|
| DHS vs FNS | 2.7430 | [1.2208; 6.1633] | 2.44 | 0.0146 |
| DHS vs CCS | 1.4394 | [1.0461; 1.9806] | 2.24 | 0.0253 |
| FNS vs CCS | 0.5248 | [0.2405; 1.1451] | -1.62 | 0.1053 |

Quantifying heterogeneity / inconsistency:
tau^2 = 0; tau = 0; I^2 = 0% [0.0%; 48.0%]

Tests of heterogeneity (within designs) and inconsistency (between designs):

| | Q | d.f. | p-value |
|---|---|---|---|
| Total | 10.53 | 19 | 0.9388 |
| Within designs | 9.51 | 18 | 0.9468 |
| Between designs | 1.02 | 1 | 0.3133 |

Details of network meta-analysis methods:
- Frequentist graph-theoretical approach
- DerSimonian-Laird estimator for tau^2
- Calculation of I^2 based on Q

## Femoral neck shortening

Fig. S7: Forest plots for femoral neck shortening from the network meta-analysis.

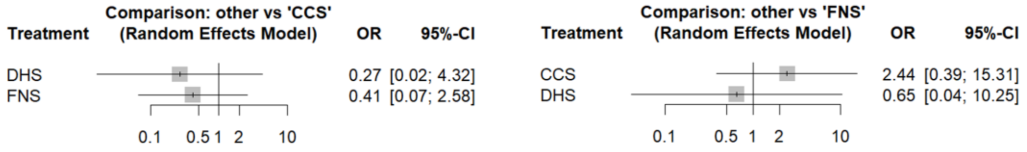


Treatment estimate:

| | MD | 95%-CI | z | p-value |
|---|---|---|---|---|
| DHS vs FNS | 0.6473 | [0.0409; 10.2504] | -0.31 | 0.7576 |
| DHS vs CCS | 0.2658 | [0.0163; 4.3237] | -0.93 | 0.3518 |
| FNS vs CCS | 0.4106 | [0.0653; 2.5818] | -0.95 | 0.3427 |

Quantifying heterogeneity / inconsistency:
tau^2 = 2.5415; tau = 1.5942; I^2 = 82.2% [54.1%; 93.1%]

Tests of heterogeneity (within designs) and inconsistency (between designs):

| | Q | d.f. | p-value |
|---|---|---|---|
| Total | 16.85 | 3 | 0.0008 |
| Within designs | 15.21 | 2 | 0.0005 |
| Between designs | 1.64 | 1 | 0.2008 |

Details of network meta-analysis methods:
- Frequentist graph-theoretical approach
- DerSimonian-Laird estimator for tau^2
- Calculation of I^2 based on Q

## Implant failure/cut-out

Fig. S9: Forest plots for implant failure/cut-out from the network meta-analysis.

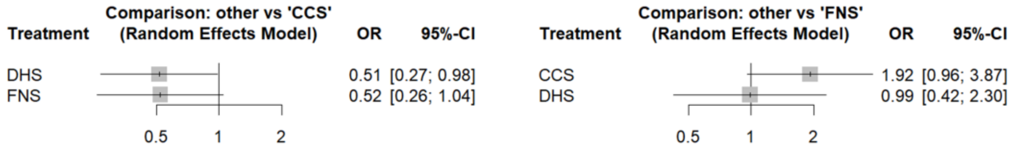


Treatment estimate:

| | MD | 95%-CI | z | p-value |
|---|---|---|---|---|
| DHS vs FNS | 0.9884 | [0.4241; 2.3036] | -0.03 | 0.9783 |
| DHS vs CCS | 0.5137 | [0.2685; 0.9826] | -2.01 | 0.0441 |
| FNS vs CCS | 0.5197 | [0.2586; 1.0443] | -1.84 | 0.0660 |

Quantifying heterogeneity / inconsistency:
tau^2 = 0.2512; tau = 0.5012; I^2 = 23% [0.0%; 56.4%]

Tests of heterogeneity (within designs) and inconsistency (between designs):

| | Q | d.f. | p-value |
|---|---|---|---|
| Total | 22.08 | 17 | 0.1815 |
| Within designs | 17.53 | 14 | 0.2292 |
| Between designs | 4.56 | 3 | 0.2072 |

Details of network meta-analysis methods:
- Frequentist graph-theoretical approach
- DerSimonian-Laird estimator for tau^2
- Calculation of I^2 based on Q

## Fracture nonunion/delayed union

Fig. S11: Forest plots for fracture nonunion/delayed union from the network meta-analysis.

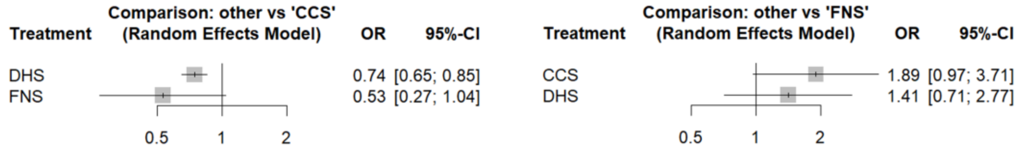


Treatment estimate:

| | MD | 95%-CI | z | p-value |
|---|---|---|---|---|
| DHS vs FNS | 1.4053 | [0.7134; 2.7686] | 0.98 | 0.3253 |
| DHS vs CCS | 0.7430 | [0.6486; 0.8512] | -4.28 | < 0.0001 |
| FNS vs CCS | 0.5287 | [0.2698; 1.0359] | -1.86 | 0.0633 |

Quantifying heterogeneity / inconsistency:
tau^2 = 0; tau = 0; I^2 = 0% [0.0%; 48.9%]

Tests of heterogeneity (within designs) and inconsistency (between designs):

| | Q | d.f. | p-value |
|---|---|---|---|
| Total | 8.49 | 18 | 0.9705 |
| Within designs | 6.89 | 15 | 0.9607 |
| Between designs | 1.60 | 3 | 0.6591 |

Details of network meta-analysis methods:
- Frequentist graph-theoretical approach
- DerSimonian-Laird estimator for tau^2
- Calculation of I^2 based on Q

## Intraoperative blood loss

Fig. S13: Forest plots for intraoperative blood loss from the network meta-analysis.

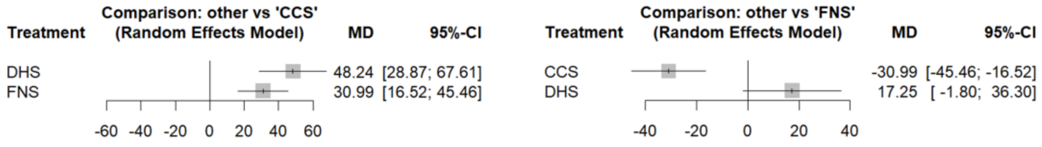


Treatment estimate:

| | MD | 95%-CI | z | p-value |
|---|---|---|---|---|
| DHS vs FNS | 17.2508 | [ -1.7969; 36.2986] | 1.78 | 0.0759 |
| DHS vs CCS | 48.2427 | [28.8743; 67.6111] | 4.88 | < 0.0001 |
| FNS vs CCS | 30.9919 | [16.5229; 45.4608] | 4.20 | < 0.0001 |

Quantifying heterogeneity / inconsistency:
tau^2 = 393.9372; tau = 19.8479; I^2 = 96.8% [95.5%; 97.7%]

Tests of heterogeneity (within designs) and inconsistency (between designs):

| | Q | d.f. | p-value |
|---|---|---|---|
| Total | 309.87 | 10 | < 0.0001 |
| Within designs | 218.84 | 7 | < 0.0001 |
| Between designs | 91.03 | 3 | < 0.0001 |

Details of network meta-analysis methods:
- Frequentist graph-theoretical approach
- DerSimonian-Laird estimator for tau^2
- Calculation of I^2 based on Q

## Operative time

Fig. S15: Forest plots for operative time from the network meta-analysis.

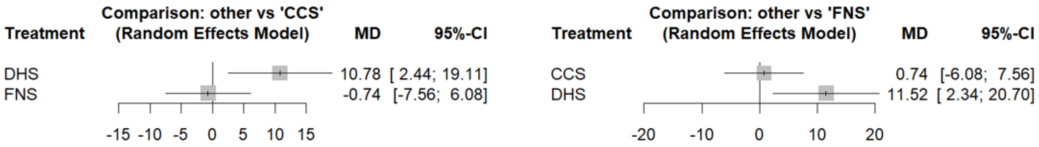


Treatment estimate:

| | MD | 95%-CI | z | p-value |
|---|---|---|---|---|
| DHS vs FNS | 11.5204 | [ 2.3430; 20.6979] | 2.46 | 0.0139 |
| DHS vs CCS | 10.7774 | [ 2.4403; 19.1144] | 2.53 | 0.0113 |
| FNS vs CCS | -0.7431 | [-7.5639; 6.0777] | -0.21 | 0.8309 |

Quantifying heterogeneity / inconsistency:
tau^2 = 123.7399; tau = 11.1238; I^2 = 94.5% [92.5%; 95.9%]

Tests of heterogeneity (within designs) and inconsistency (between designs):

| | Q | d.f. | p-value |
|---|---|---|---|
| Total | 289.53 | 16 | < 0.0001 |
| Within designs | 228.54 | 13 | < 0.0001 |
| Between designs | 60.99 | 3 | < 0.0001 |

Details of network meta-analysis methods:
- Frequentist graph-theoretical approach
- DerSimonian-Laird estimator for tau^2
- Calculation of I^2 based on Q

## Fracture healing time

Fig. S17: Forest plots for fracture healing time from the network meta-analysis.

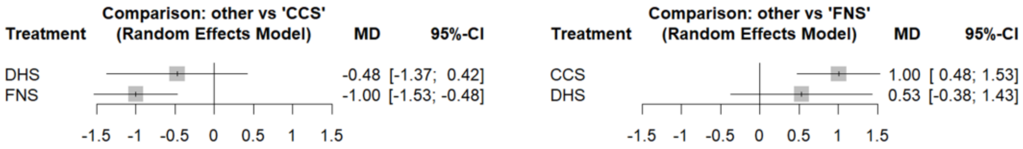


Treatment estimate:

| | MD | 95%-CI | z | p-value |
|---|---|---|---|---|
| DHS vs FNS | 0.5280 | [-0.3754; 1.4313] | 1.15 | 0.2520 |
| DHS vs CCS | -0.4751 | [-1.3734; 0.4231] | -1.04 | 0.2999 |
| FNS vs CCS | -1.0031 | [-1.5308; -0.4754] | -3.73 | 0.0002 |

Quantifying heterogeneity / inconsistency:
tau^2 = 0.3637; tau = 0.6031; I^2 = 94.2% [90.0%; 96.7%]

Tests of heterogeneity (within designs) and inconsistency (between designs):

| | Q | d.f. | p-value |
|---|---|---|---|
| Total | 86.77 | 5 | < 0.0001 |
| Within designs | 73.13 | 4 | < 0.0001 |
| Between designs | 13.64 | 1 | 0.0002 |

Details of network meta-analysis methods:
- Frequentist graph-theoretical approach
- DerSimonian-Laird estimator for tau^2
- Calculation of I^2 based on Q

**Fig. S18: Funnel plots for primary and secondary outcomes.**

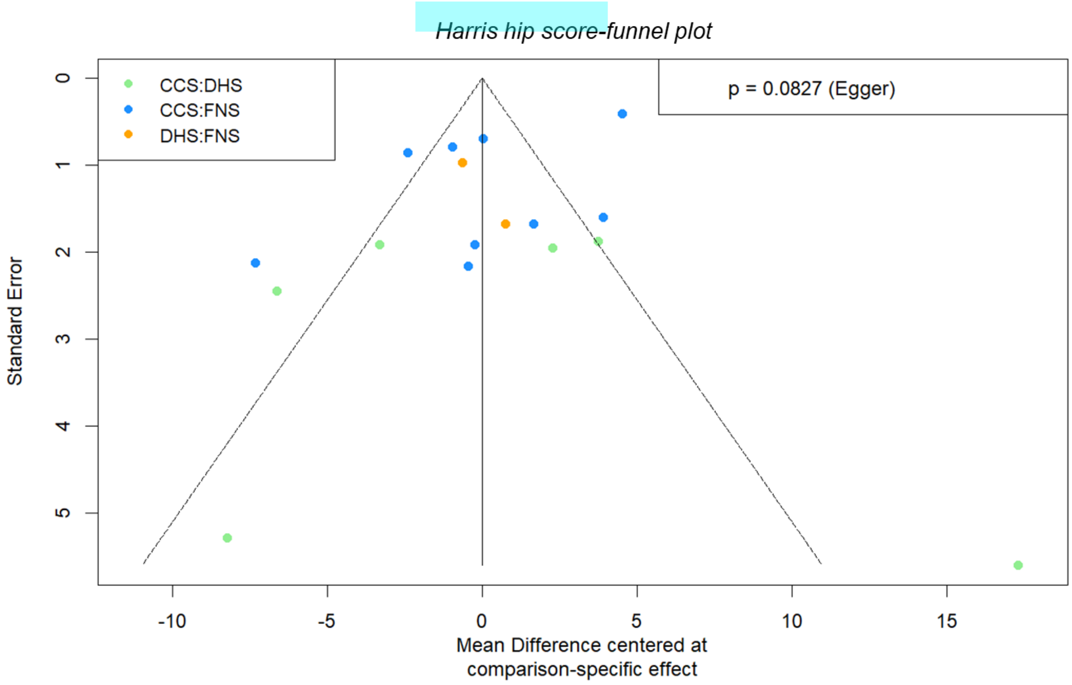


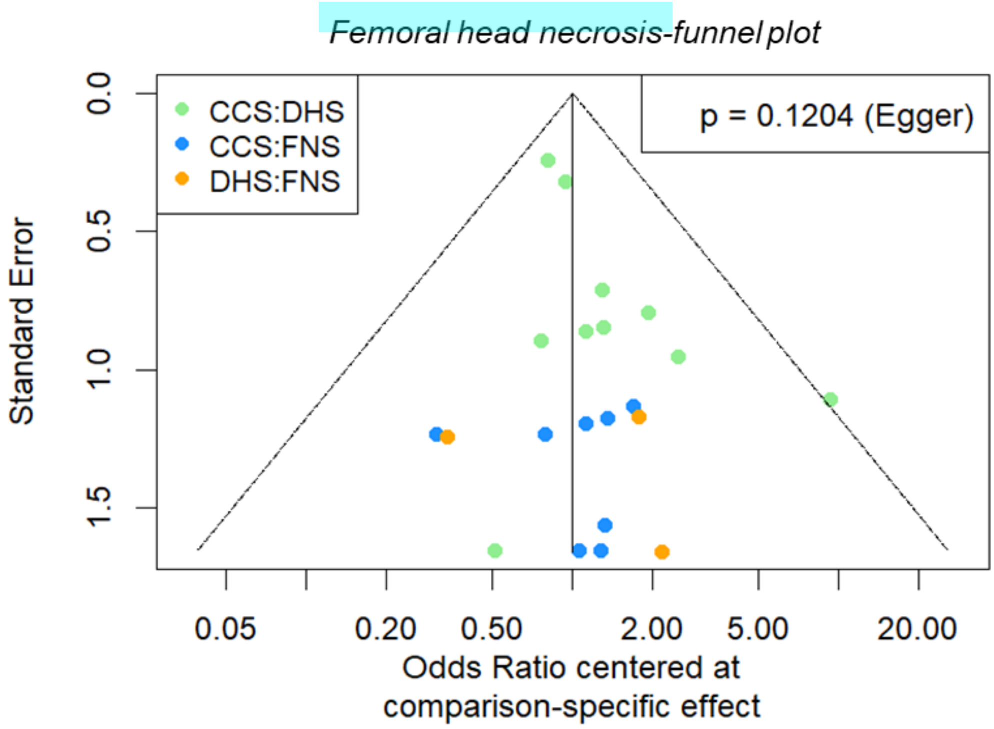

*Femoral neck shortening-funnel plot*

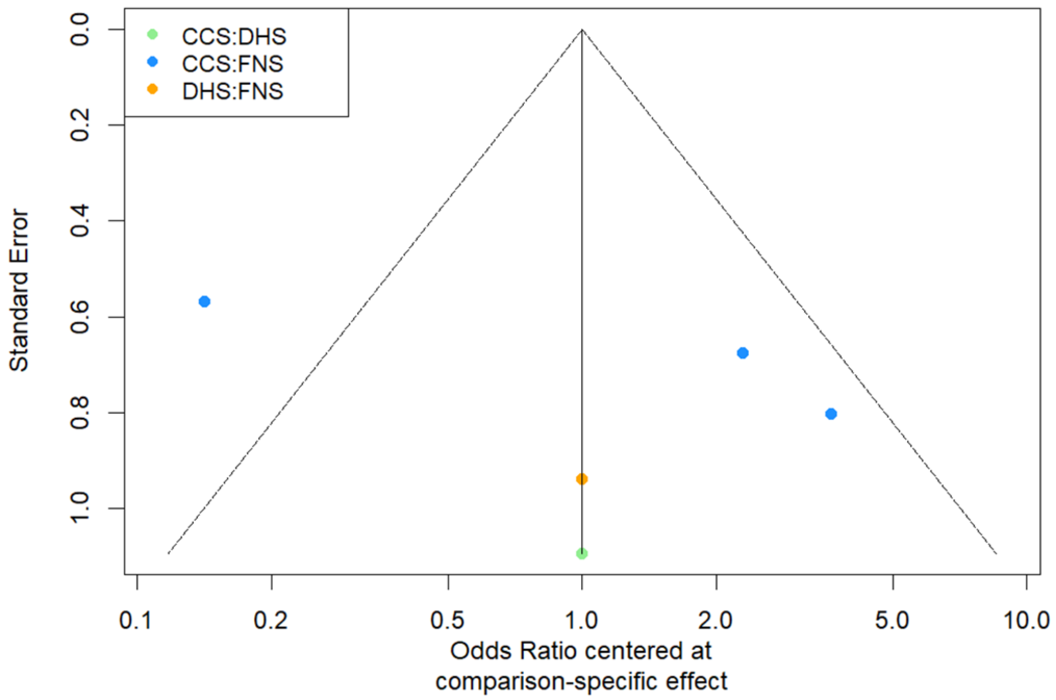


*Implant failure/cutout-funnel plot*

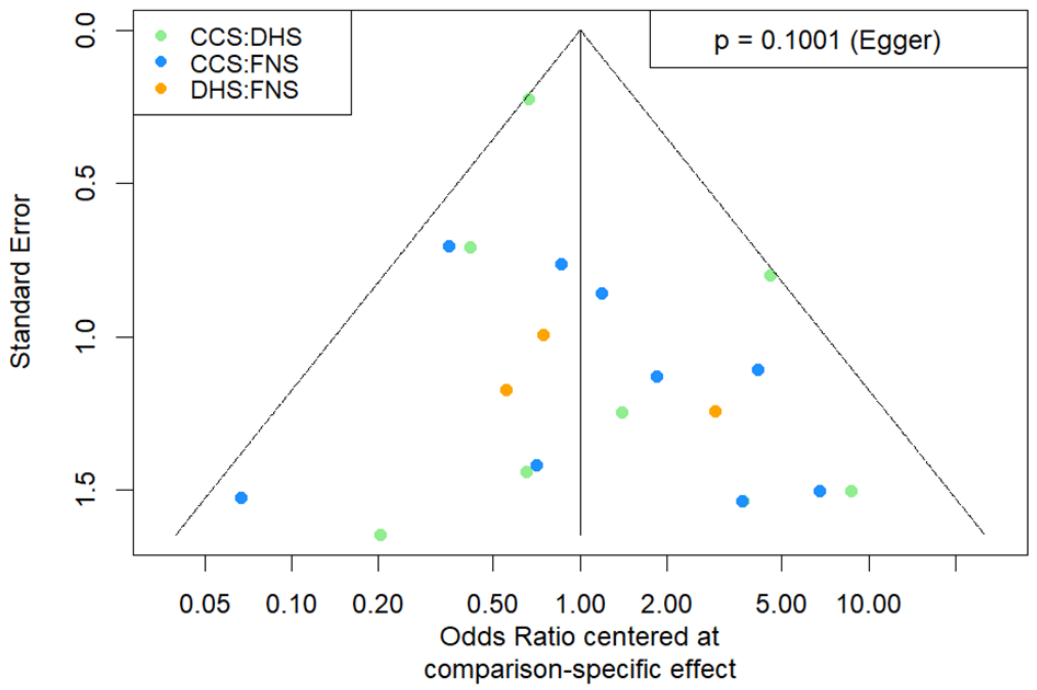

*Fracture nonunion/delayed union-funnel plot*

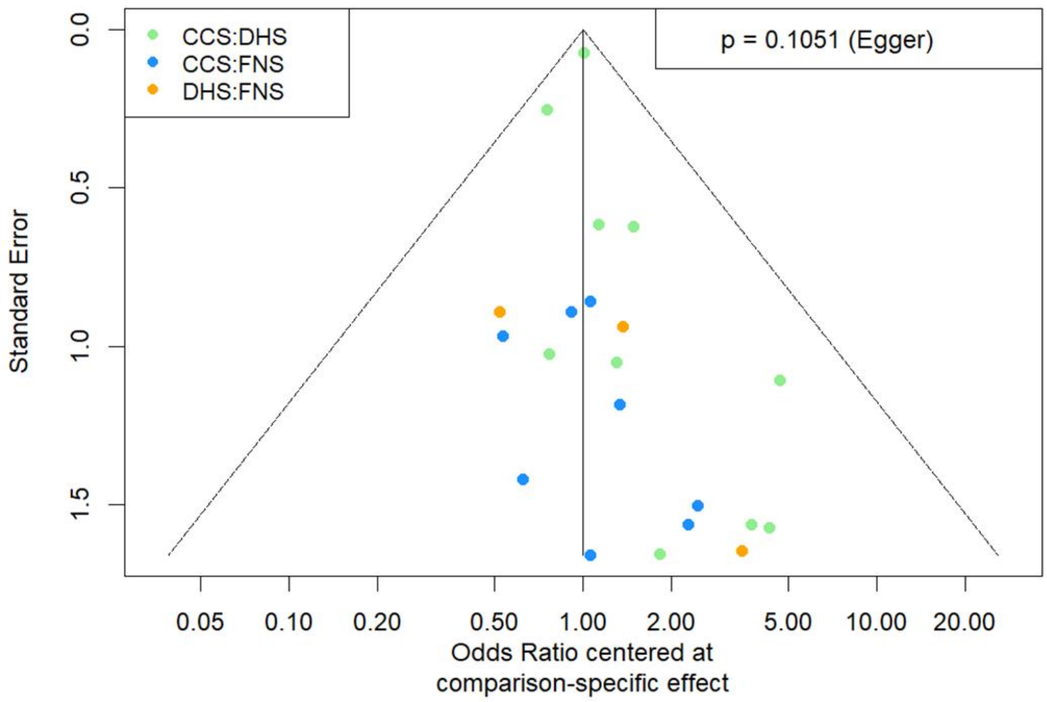


*Intraoperative blood loss-funnel plot*

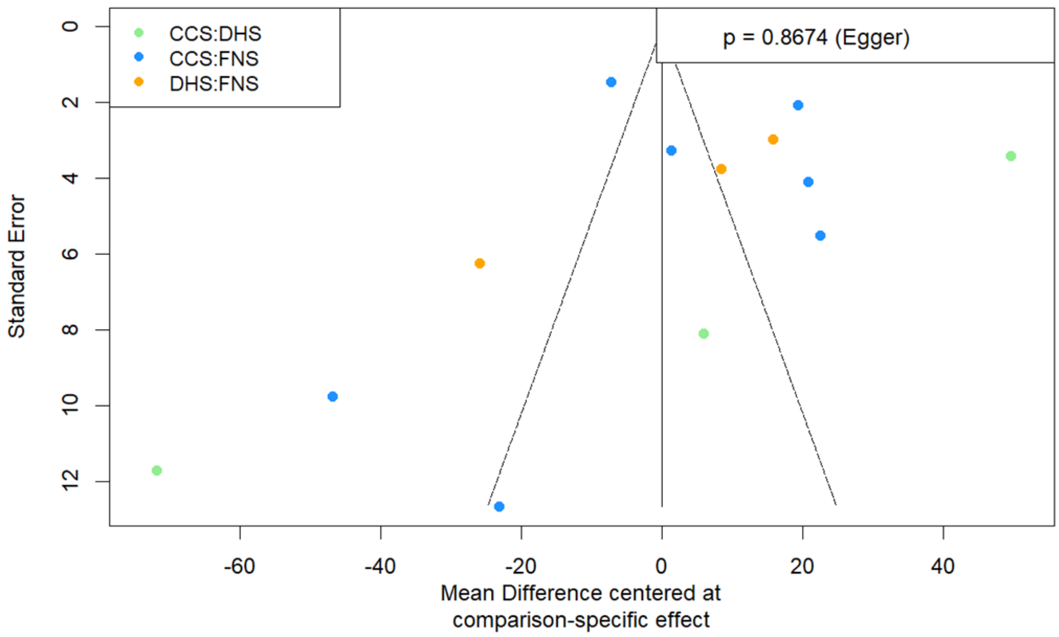

*Operation time-funnel plot*

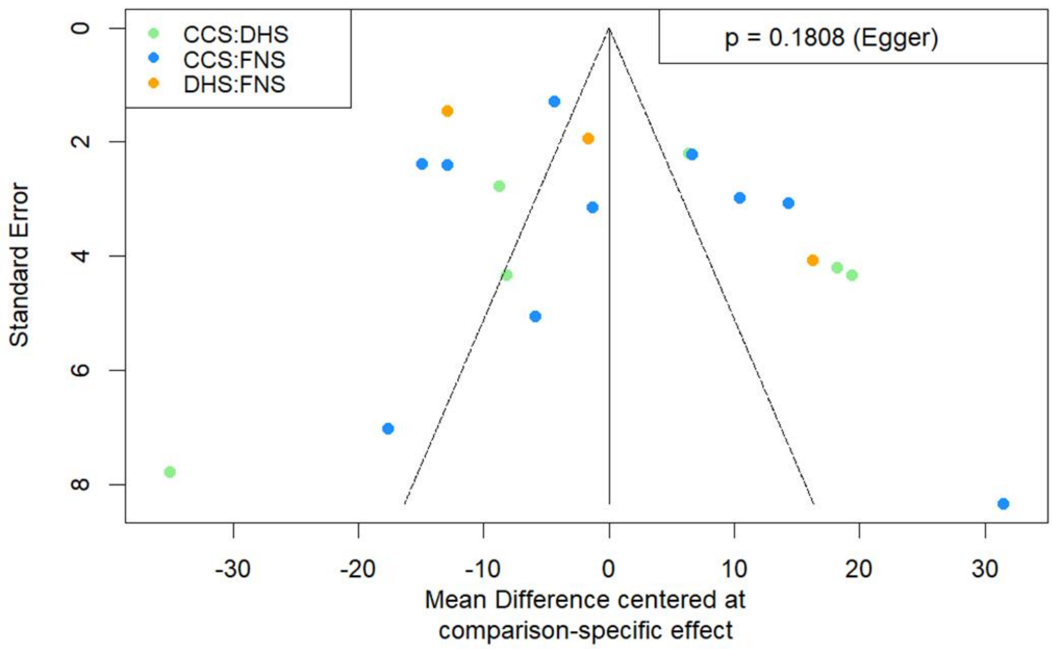


*Fracture healing time-funnel plot*

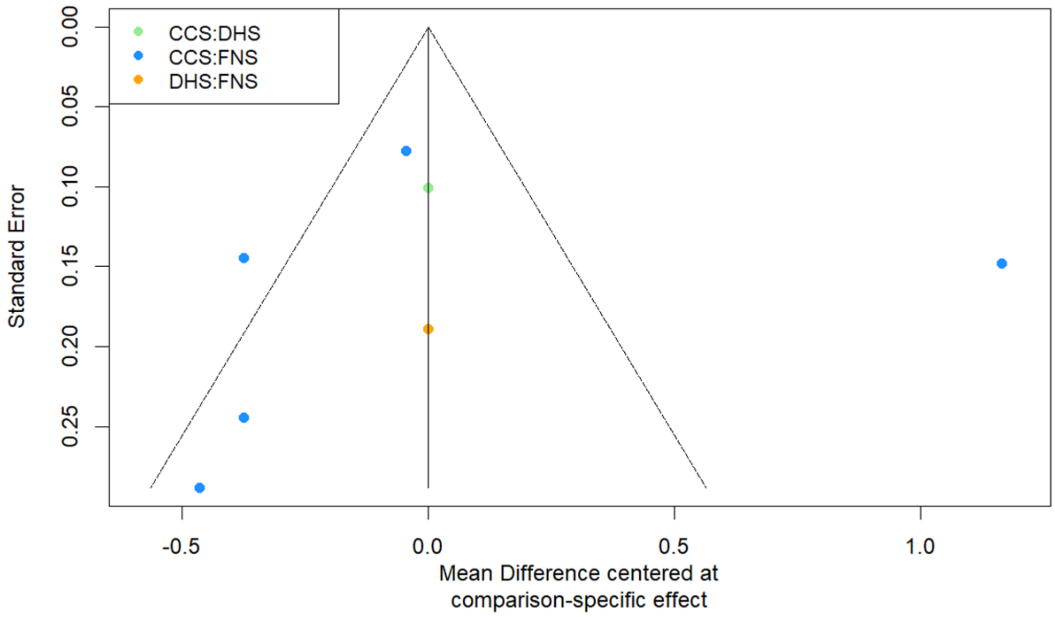

## Node-Splitting Analysis

The following plots evaluate the inconsistency between direct and indirect evidence in the network. A non-significant p-value (> 0.05) suggests good consistency.

### Harris Hip Score

Fig. S19: Node-splitting analysis for Harris Hip Score in network meta-analysis: evaluating inconsistency between direct and indirect evidence.

Random effects model:

| comparison | k | prop | nma | direct | indir. | Diff | z | p-value |
|---|---|---|---|---|---|---|---|---|
| DHS:CCS | 6 | 0.79 | 2.4514 | 2.3810 | 2.7141 | -0.3331 | -0.08 | 0.9339 |
| FNS:CCS | 9 | 0.91 | 3.7853 | 3.5396 | 6.2828 | -2.7432 | -0.65 | 0.5133 |
| DHS:FNS | 2 | 0.46 | -1.3339 | -1.7515 | -0.9726 | -0.7789 | -0.22 | 0.8291 |

Legend:
comparison - Treatment comparison
k - Number of studies providing direct evidence
prop - Direct evidence proportion
nma - Estimated treatment effect (MD) in network meta-analysis
direct - Estimated treatment effect (MD) derived from direct evidence
indir. - Estimated treatment effect (MD) derived from indirect evidence
Diff - Difference between direct and indirect treatment estimates
z - z-value of test for disagreement (direct versus indirect)
p-value - p-value of test for disagreement (direct versus indirect)

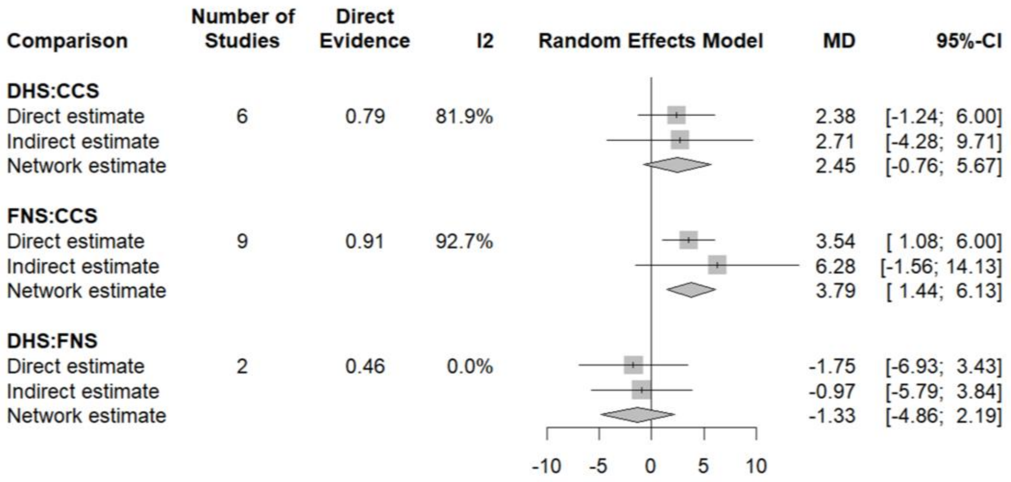

## Femoral head necrosis

Fig. S20: Node-splitting analysis for femoral head necrosis in network meta-analysis: evaluating inconsistency between direct and indirect evidence.

Random effects model:

| comparison | k | prop | nma | direct | indir. | RoR | z | p-value |
|---|---|---|---|---|---|---|---|---|
| DHS:CCS | 10 | 0.97 | 1.4394 | 1.4841 | 0.5961 | 2.4897 | 1.01 | 0.3133 |
| FNS:CCS | 8 | 0.74 | 0.5248 | 0.4130 | 1.0282 | 0.4017 | -1.01 | 0.3133 |
| DHS:FNS | 3 | 0.30 | 2.7430 | 1.4434 | 3.5938 | 0.4017 | -1.01 | 0.3133 |

Legend:
comparison - Treatment comparison
k - Number of studies providing direct evidence
prop - Direct evidence proportion
nma - Estimated treatment effect (OR) in network meta-analysis
direct - Estimated treatment effect (OR) derived from direct evidence
indir. - Estimated treatment effect (OR) derived from indirect evidence
RoR - Ratio of Ratios (direct versus indirect)
z - z-value of test for disagreement (direct versus indirect)
p-value - p-value of test for disagreement (direct versus indirect)

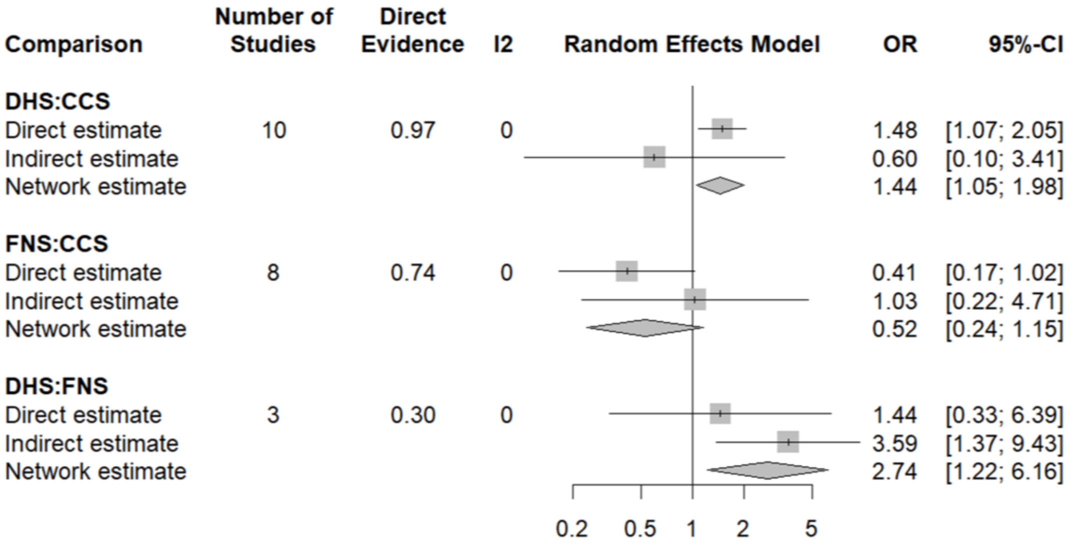

**Femoral neck shortening**

Fig. S21: Node-splitting analysis for femoral neck shortening in network meta-analysis: evaluating inconsistency between direct and indirect evidence.

Random effects model:

| comparison | k | prop | nma | direct | indir. | RoR | z | p-value |
|---|---|---|---|---|---|---|---|---|
| DHS:CCS | 1 | 0.54 | 0.2658 | 0.1289 | 0.6259 | 0.2060 | -0.55 | 0.5802 |
| FNS:CCS | 3 | 0.88 | 0.4106 | 0.4987 | 0.1027 | 4.8549 | 0.55 | 0.5802 |
| DHS:FNS | 1 | 0.58 | 0.6473 | 1.2551 | 0.2585 | 4.8549 | 0.55 | 0.5802 |

Legend:
comparison - Treatment comparison
k - Number of studies providing direct evidence
prop - Direct evidence proportion
nma - Estimated treatment effect (OR) in network meta-analysis
direct - Estimated treatment effect (OR) derived from direct evidence
indir. - Estimated treatment effect (OR) derived from indirect evidence
RoR - Ratio of Ratios (direct versus indirect)
z - z-value of test for disagreement (direct versus indirect)
p-value - p-value of test for disagreement (direct versus indirect)

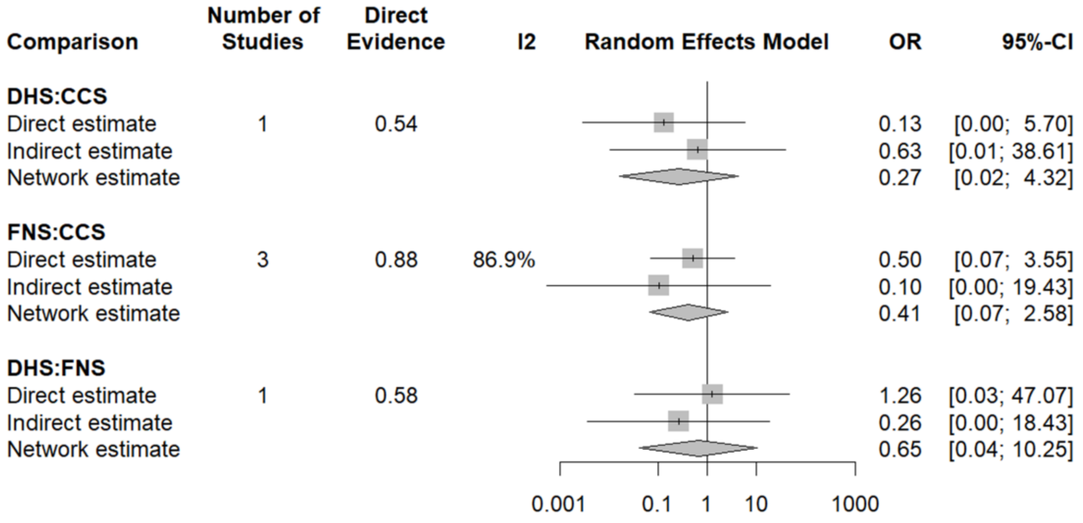

## Implant failure/cut-out

Fig. S22: Node-splitting analysis for implant failure/cut-out in network meta-analysis: evaluating inconsistency between direct and indirect evidence.

Random effects model:

| comparison | k | prop | nma | direct | indir. | RoR | z | p-value |
|---|---|---|---|---|---|---|---|---|
| DHS:CCS | 8 | 0.86 | 0.5137 | 0.6089 | 0.1772 | 3.4363 | 1.29 | 0.1986 |
| FNS:CCS | 9 | 0.82 | 0.5197 | 0.4677 | 0.8497 | 0.5505 | -0.64 | 0.5227 |
| DHS:FNS | 3 | 0.37 | 0.9884 | 0.5744 | 1.3559 | 0.4236 | -0.96 | 0.3373 |

Legend:
comparison - Treatment comparison
k - Number of studies providing direct evidence
prop - Direct evidence proportion
nma - Estimated treatment effect (OR) in network meta-analysis
direct - Estimated treatment effect (OR) derived from direct evidence
indir. - Estimated treatment effect (OR) derived from indirect evidence
RoR - Ratio of Ratios (direct versus indirect)
z - z-value of test for disagreement (direct versus indirect)
p-value - p-value of test for disagreement (direct versus indirect)

| Comparison | Number of Studies | Direct Evidence | I2 | OR | 95%-CI |
|---|---|---|---|---|---|
| **DHS:CCS** | | | | | |
| Direct estimate | 8 | 0.86 | 34.5% | 0.61 | [0.30; 1.22] |
| Indirect estimate | | | | 0.18 | [0.03; 1.02] |
| Network estimate | | | | 0.51 | [0.27; 0.98] |
| **FNS:CCS** | | | | | |
| Direct estimate | 9 | 0.82 | 17.9% | 0.47 | [0.22; 1.01] |
| Indirect estimate | | | | 0.85 | [0.16; 4.47] |
| Network estimate | | | | 0.52 | [0.26; 1.04] |
| **DHS:FNS** | | | | | |
| Direct estimate | 3 | 0.37 | 0.0% | 0.57 | [0.14; 2.32] |
| Indirect estimate | | | | 1.36 | [0.47; 3.93] |
| Network estimate | | | | 0.99 | [0.42; 2.30] |

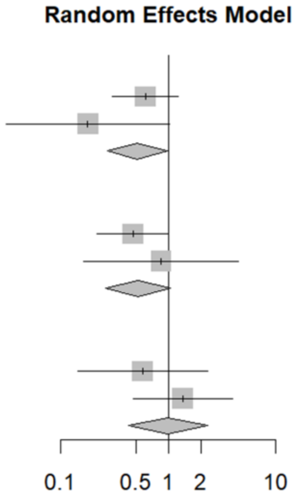

**Fracture nonunion/delayed union**

Fig. S23: Node-splitting analysis for fracture nonunion/delayed union in network meta-analysis: evaluating inconsistency between direct and indirect evidence.

Random effects model:

| comparison | k | prop | nma | direct | indir. | RoR | z | p-value |
|---|---|---|---|---|---|---|---|---|
| DHS:CCS | 10 | 0.99 | 0.7430 | 0.7478 | 0.3394 | 2.2032 | 1.02 | 0.3068 |
| FNS:CCS | 8 | 0.72 | 0.5287 | 0.4138 | 0.9886 | 0.4186 | -1.14 | 0.2537 |
| DHS:FNS | 3 | 0.33 | 1.4053 | 0.9159 | 1.7363 | 0.5275 | -0.87 | 0.3844 |

Legend:
comparison - Treatment comparison
k - Number of studies providing direct evidence
prop - Direct evidence proportion
nma - Estimated treatment effect (OR) in network meta-analysis
direct - Estimated treatment effect (OR) derived from direct evidence
indir. - Estimated treatment effect (OR) derived from indirect evidence
RoR - Ratio of Ratios (direct versus indirect)
z - z-value of test for disagreement (direct versus indirect)
p-value - p-value of test for disagreement (direct versus indirect)

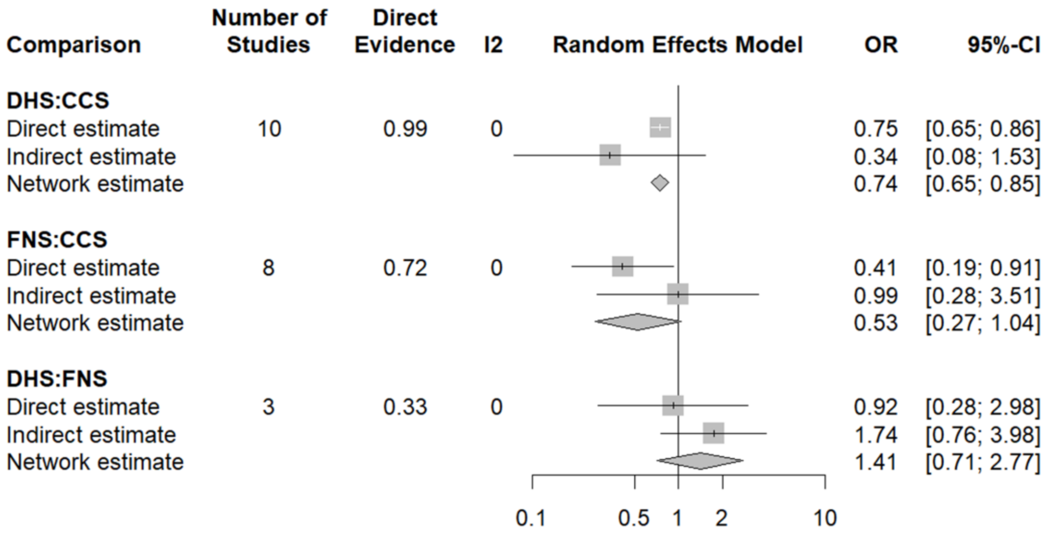

**Intraoperative blood loss**

Fig. S24: Node-splitting analysis for intraoperative blood loss in network meta-analysis: evaluating inconsistency between direct and indirect evidence.

Random effects model:

| comparison | k | prop | nma | direct | indir. | Diff | z | p-value |
|---|---|---|---|---|---|---|---|---|
| DHS:CCS | 3 | 0.64 | 48.2427 | 60.7471 | 26.2910 | 34.4561 | 1.68 | 0.0936 |
| FNS:CCS | 7 | 0.88 | 30.9919 | 22.5883 | 92.6093 | -70.0211 | -3.08 | 0.0021 |
| DHS:FNS | 3 | 0.68 | 17.2508 | 0.9467 | 52.5587 | -51.6120 | -2.47 | 0.0136 |

Legend:
comparison - Treatment comparison
k - Number of studies providing direct evidence
prop - Direct evidence proportion
nma - Estimated treatment effect (MD) in network meta-analysis
direct - Estimated treatment effect (MD) derived from direct evidence
indir. - Estimated treatment effect (MD) derived from indirect evidence
Diff - Difference between direct and indirect treatment estimates
z - z-value of test for disagreement (direct versus indirect)
p-value - p-value of test for disagreement (direct versus indirect)

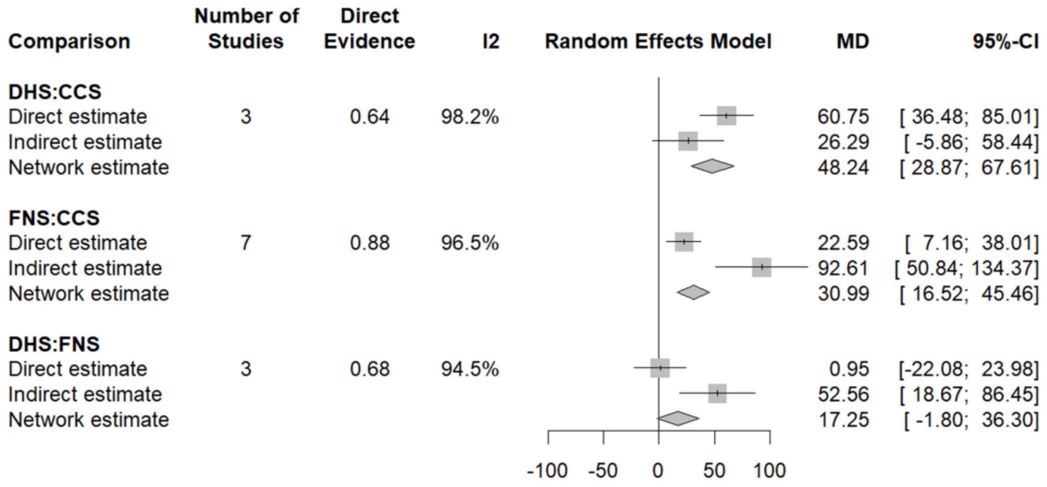

**Operative time**

Fig. S25: Node-splitting analysis for operative time in network meta-analysis: evaluating inconsistency between direct and indirect evidence.

Random effects model:

| comparison | k | prop | nma | direct | indir. | Diff | z | p-value |
|---|---|---|---|---|---|---|---|---|
| DHS:CCS | 6 | 0.76 | 10.7774 | 14.3748 | -0.5264 | 14.9012 | 1.50 | 0.1338 |
| FNS:CCS | 10 | 0.86 | -0.7431 | -2.4630 | 10.2454 | -12.7084 | -1.25 | 0.2116 |
| DHS:FNS | 3 | 0.50 | 11.5204 | 7.4948 | 15.5852 | -8.0905 | -0.86 | 0.3876 |

Legend:
comparison - Treatment comparison
k - Number of studies providing direct evidence
prop - Direct evidence proportion
nma - Estimated treatment effect (MD) in network meta-analysis
direct - Estimated treatment effect (MD) derived from direct evidence
indir. - Estimated treatment effect (MD) derived from indirect evidence
Diff - Difference between direct and indirect treatment estimates
z - z-value of test for disagreement (direct versus indirect)
p-value - p-value of test for disagreement (direct versus indirect)

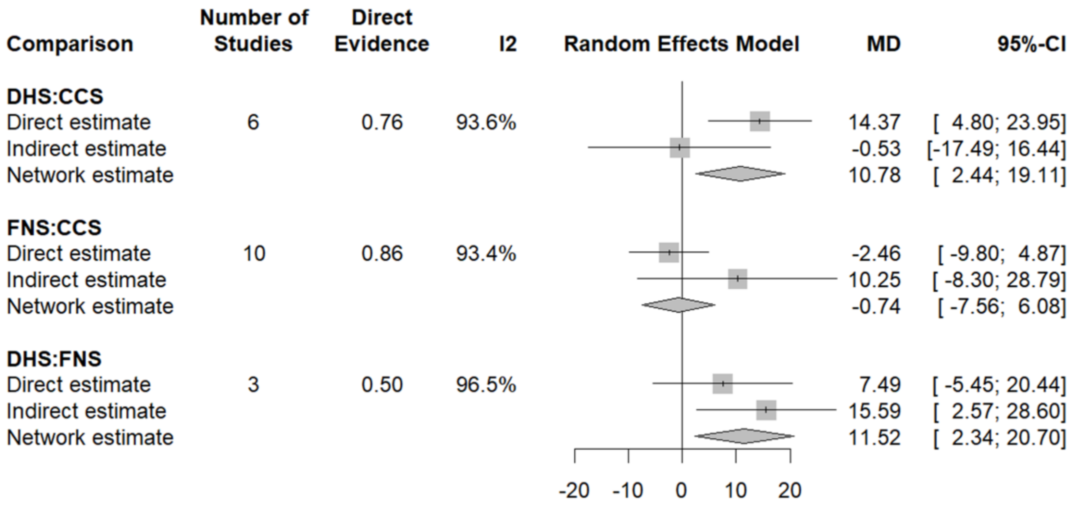

**Fracture healing time**

Fig. S26: Node-splitting analysis for fracture healing time in network meta-analysis: evaluating inconsistency between direct and indirect evidence.

Random effects model:

| comparison | k | prop | nma | direct | indir. | Diff | z | p-value |
|---|---|---|---|---|---|---|---|---|
| DHS:CCS | 1 | 0.56 | -0.4751 | -0.1400 | -0.9048 | 0.7648 | 0.83 | 0.4077 |
| FNS:CCS | 5 | 0.91 | -1.0031 | -1.0748 | -0.3100 | -0.7648 | -0.83 | 0.4077 |
| DHS:FNS | 1 | 0.53 | 0.5280 | 0.1700 | 0.9348 | -0.7648 | -0.83 | 0.4077 |

Legend:
comparison - Treatment comparison
k - Number of studies providing direct evidence
prop - Direct evidence proportion
nma - Estimated treatment effect (MD) in network meta-analysis
direct - Estimated treatment effect (MD) derived from direct evidence
indir. - Estimated treatment effect (MD) derived from indirect evidence
Diff - Difference between direct and indirect treatment estimates
z - z-value of test for disagreement (direct versus indirect)
p-value - p-value of test for disagreement (direct versus indirect)

| Comparison | Number of Studies | Direct Evidence | I2 | Random Effects Model | MD | 95%-CI |
|---|---|---|---|---|---|---|
| **DHS:CCS** | | | | | | |
| Direct estimate | 1 | 0.56 | | | -0.14 | [-1.34; 1.06] |
| Indirect estimate | | | | | -0.90 | [-2.26; 0.45] |
| Network estimate | | | | | -0.48 | [-1.37; 0.42] |
| **FNS:CCS** | | | | | | |
| Direct estimate | 5 | 0.91 | 94.5% | | -1.07 | [-1.63; -0.52] |
| Indirect estimate | | | | | -0.31 | [-2.03; 1.41] |
| Network estimate | | | | | -1.00 | [-1.53; -0.48] |
| **DHS:FNS** | | | | | | |
| Direct estimate | 1 | 0.53 | | | 0.17 | [-1.07; 1.41] |
| Indirect estimate | | | | | 0.93 | [-0.39; 2.26] |
| Network estimate | | | | | 0.53 | [-0.38; 1.43] |

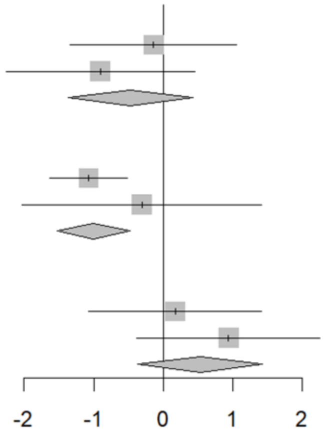

## Sensitivity and exploratory analyses

This section presents the results of leave-one-out sensitivity analysis, subgroup analyses, and meta-regression based on age, in order to assess the robustness of our findings and to explore potential sources of heterogeneity.

### Harris Hip Score

Fig. S27: Sensitivity analysis for Harris Hip Score

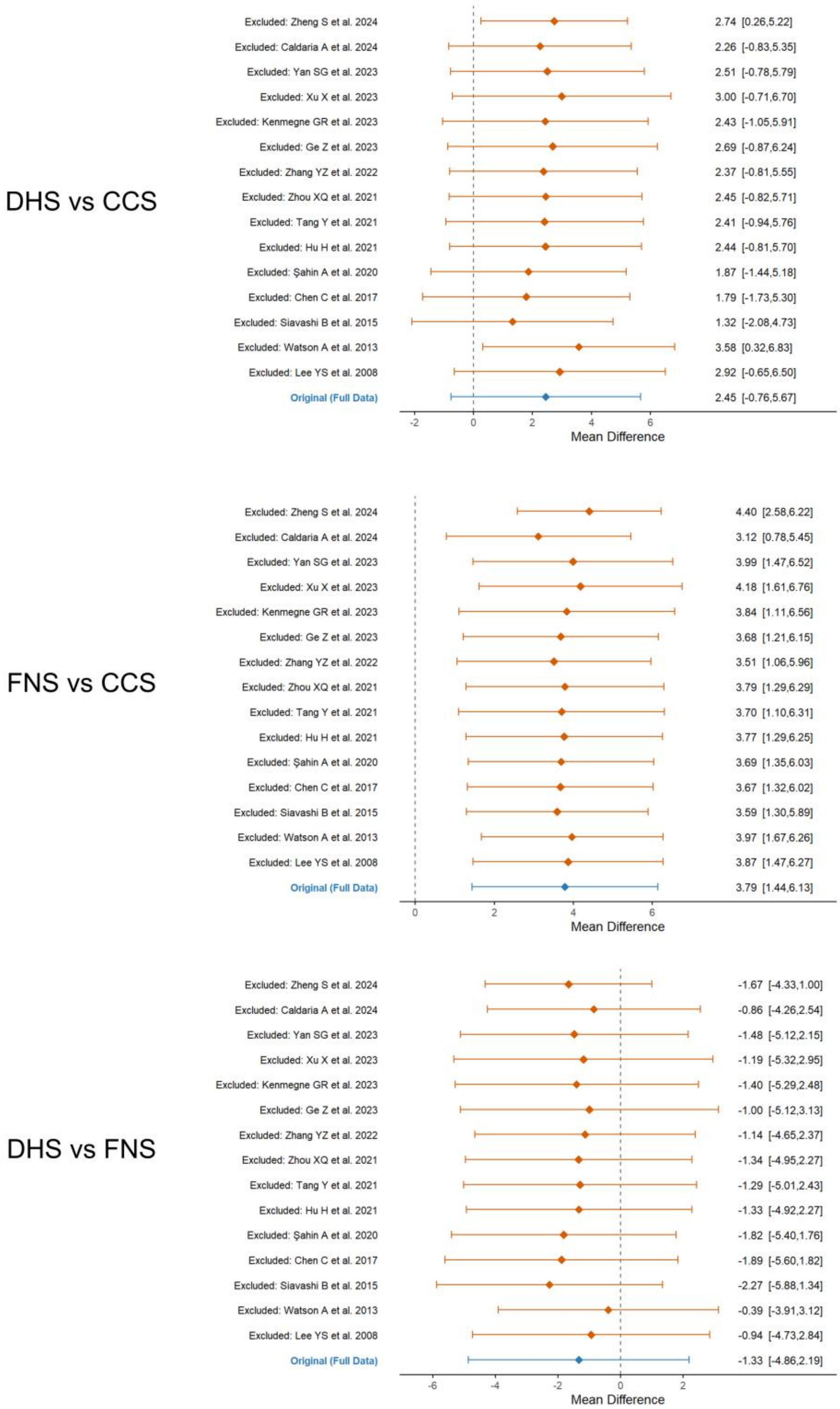

## Femoral head necrosis

Fig. S28: Sensitivity analysis for femoral head necrosis

DHS vs CCS

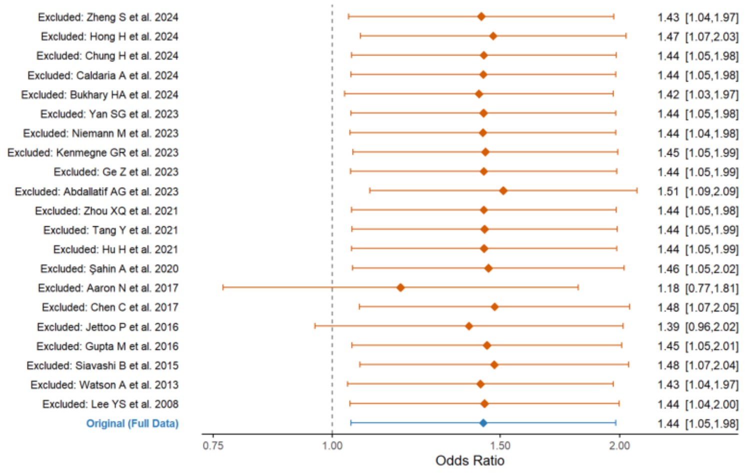


FNS vs CCS

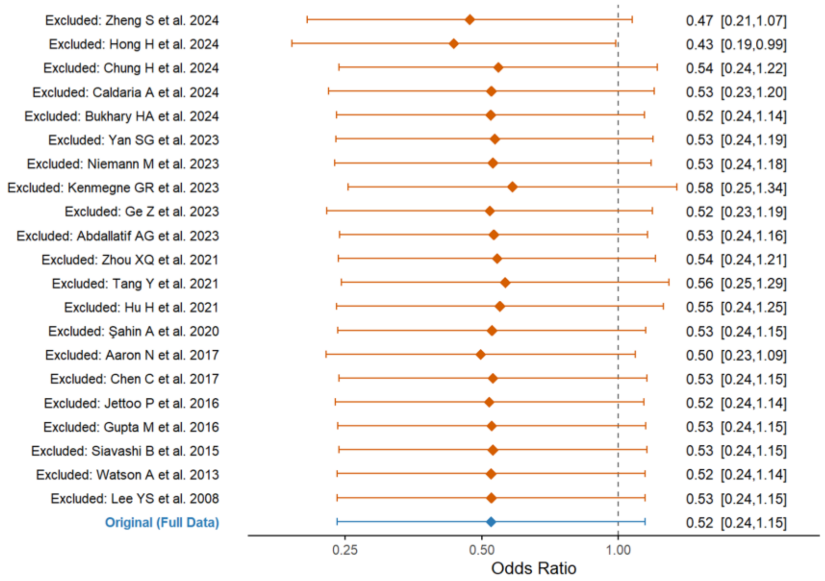


DHS vs FNS

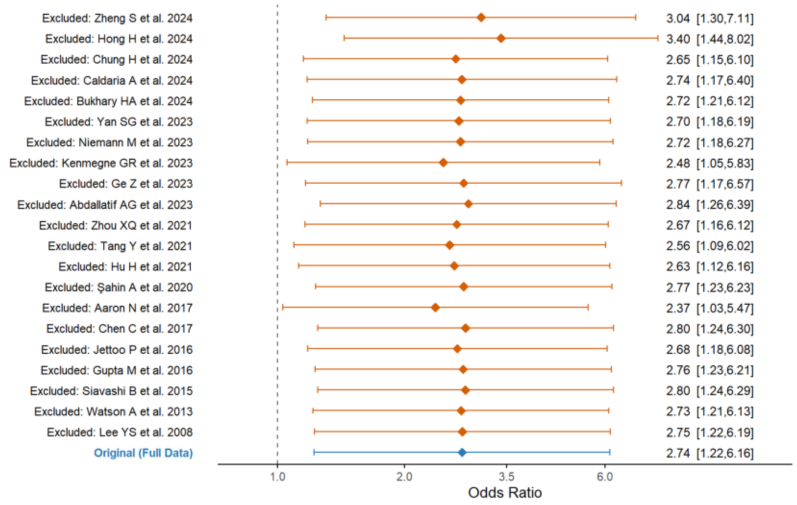

## Implant failure/cut-out

Fig. S29: Sensitivity analysis for implant failure/cut-out

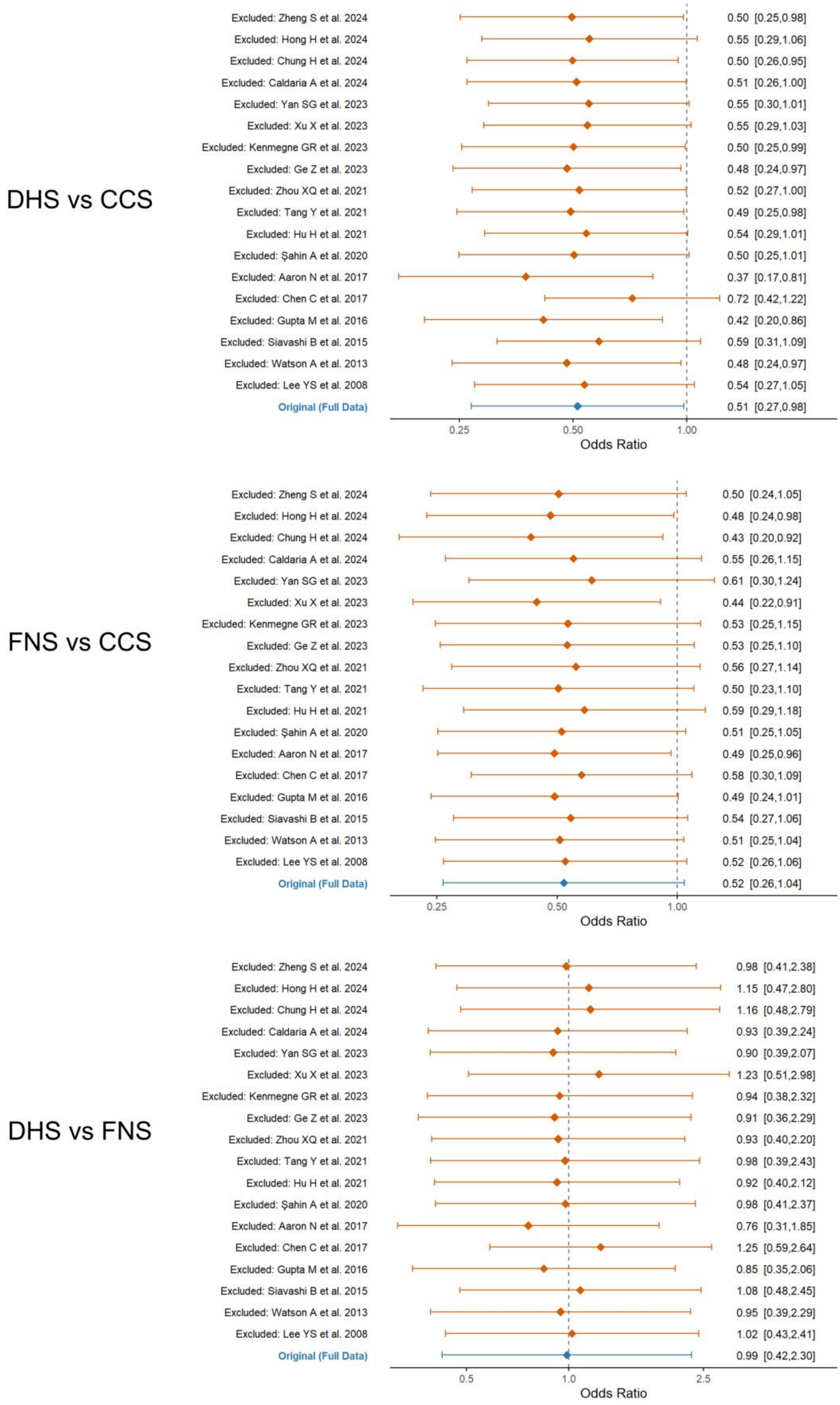

## Fracture nonunion/delayed union

Fig. S30: Sensitivity analysis for fracture nonunion/delayed union

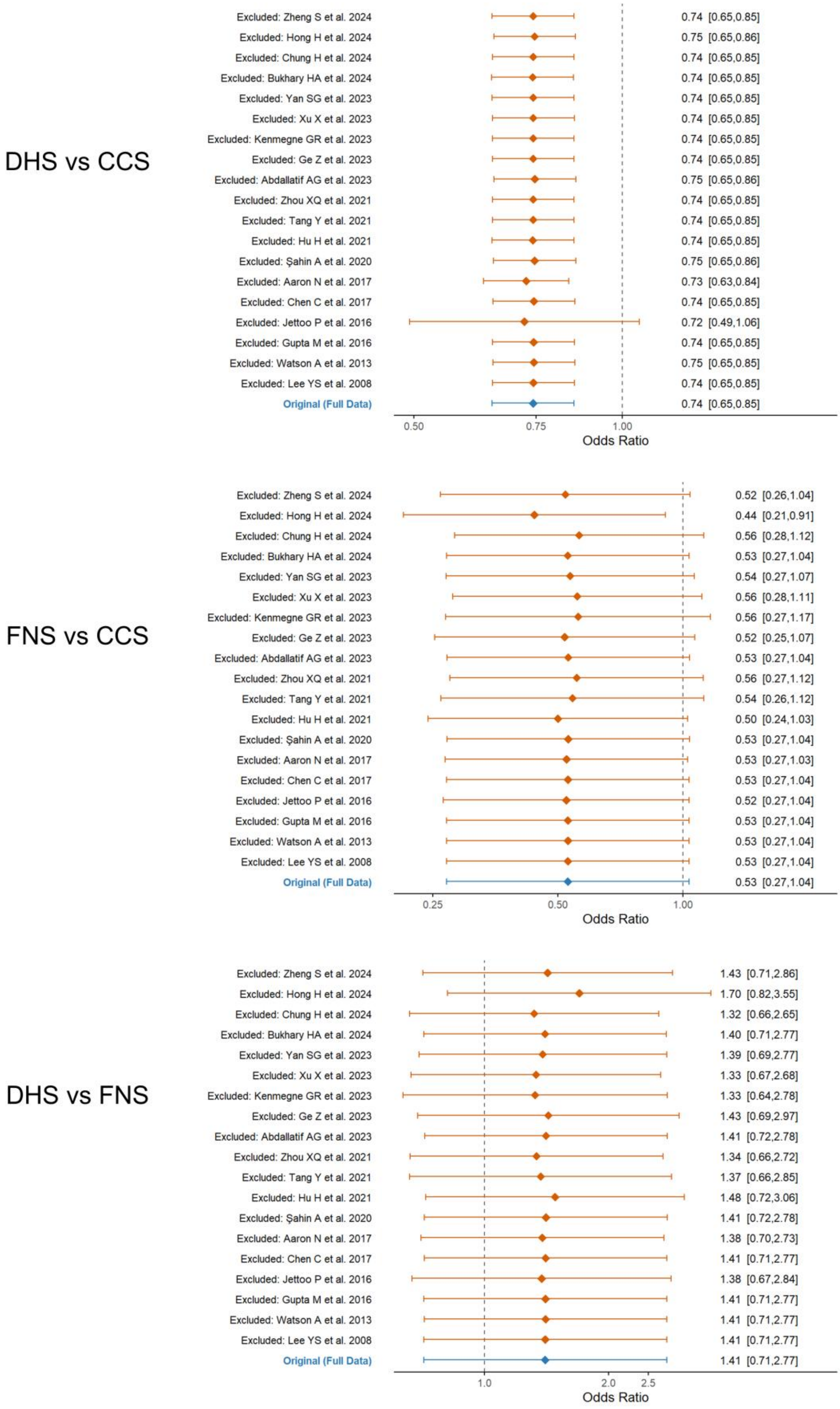

## Intraoperative blood loss

Fig. S31: Sensitivity analysis for intraoperative blood loss

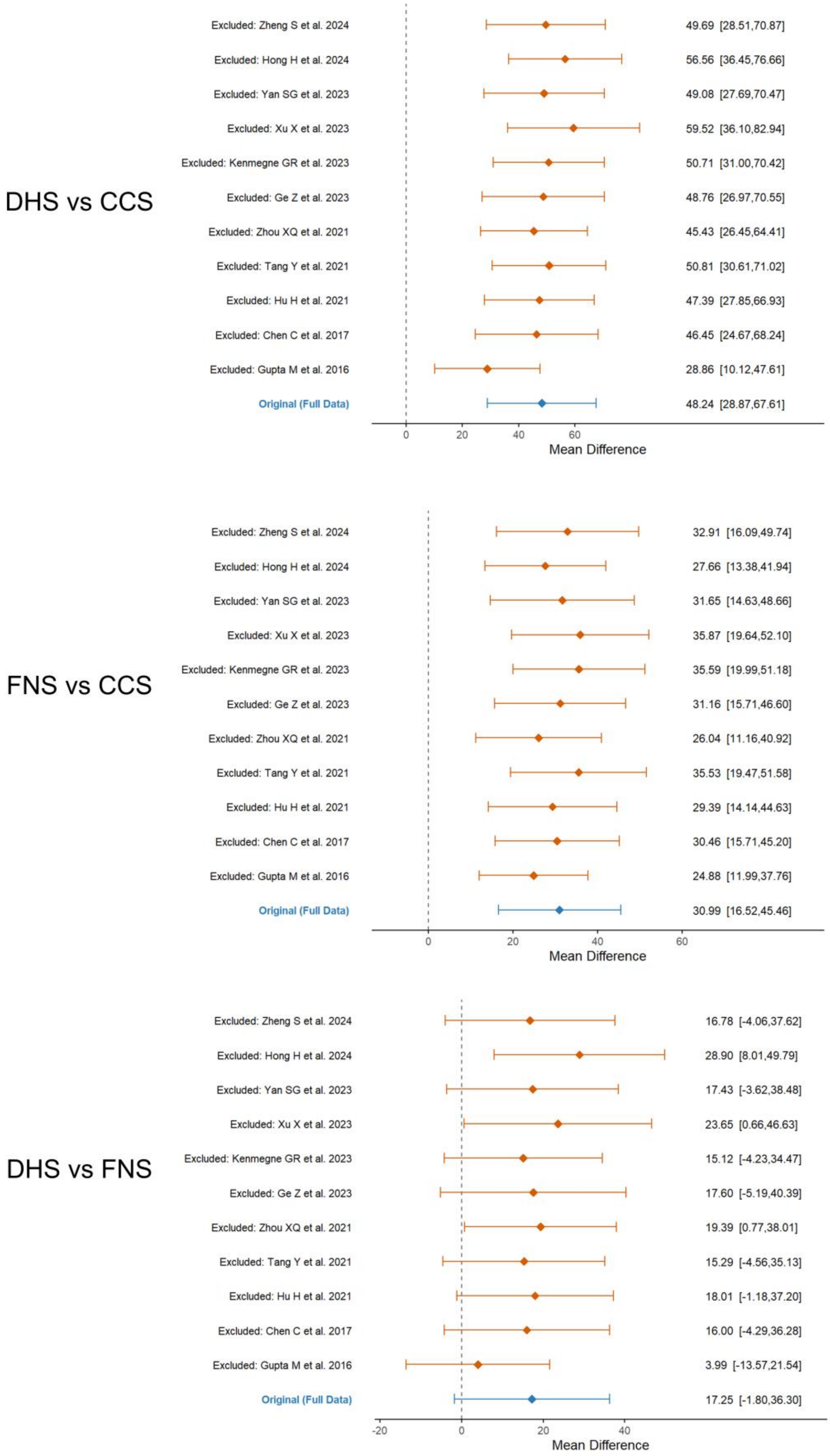

## Operative time

Fig. S32: Sensitivity analysis for operative time

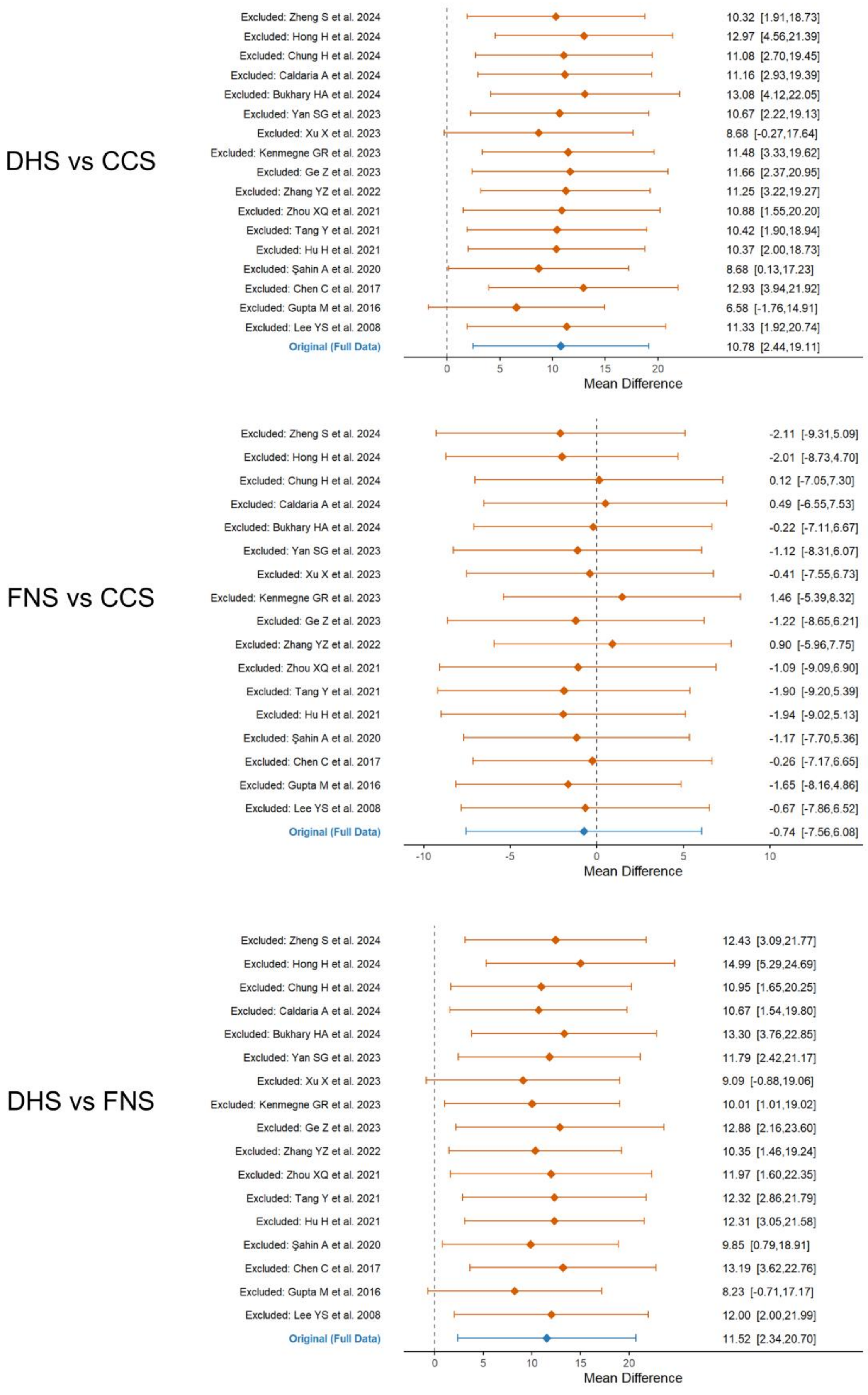

## Results of Subgroup Analyses

**Table S3: Results of Subgroup Analyses**

| Subgroup and outcomes | Number of studies | Treatment comparisons | MD(95%CI) | $I^2$ | P-Score |
|---|---|---|---|---|---|
| **Fracture type** | | | | | |
| Harris Hip Score | **Garden I-IV/II-IV: 10** | DHS vs CCS | 3.21 [0.42, 5.99] | | **0.02*** |
| | Garden I-II: 2 | FNS vs CCS | 4.54 [2.83, 6.25] | 70.9% | **<0.0001*** |
| | Garden III-IV: 2 | DHS vs FNS | -1.34 [-4.06, 1.38] | | 0.34 |
| Intraoperative blood loss(ml) | **Garden I-IV/II-IV: 9** | DHS vs CCS | 46.58 [25.72, 67.44] | | **<0.0001*** |
| | Garden I-II: 0 | FNS vs CCS | 27.29 [9.76, 44.82] | 97.2% | **0.002*** |
| | Garden III-IV: 1 | DHS vs FNS | 19.29 [-1.17, 39.75] | | 0.06 |
| Operation time(min) | **Garden I-IV/II-IV: 13** | DHS vs CCS | 13.74 [1.81, 25.67] | | **0.02*** |
| | Garden I-II: 2 | FNS vs CCS | -1.79 [-11.12, 7.54] | 95.4% | 0.71 |
| | Garden III-IV: 1 | DHS vs FNS | 15.53 [3.20, 27.86] | | **0.01*** |
| **Study type** | | | | | |
| Harris Hip Score | **CS: 13** | DHS vs CCS | 2.54 [-0.93, 6.00] | | 0.15 |
| | RCT: 2 | FNS vs CCS | 3.79 [1.54, 6.04] | 90.2% | **0.001*** |
| | | DHS vs FNS | -1.25 [-4.86, 2.36] | | 0.50 |
| Intraoperative blood loss(ml) | **CS: 9** | DHS vs CCS | 36.10 [16.51, 55.68] | | **0.0003*** |
| | RCT: 2 | FNS vs CCS | 22.89 [10.33, 35.44] | 96% | **0.0004*** |
| | | DHS vs FNS | 13.21 [6.17, 32.60] | | 0.18 |
| Operation time(min) | **CS: 15** | DHS vs CCS | 8.66 [0.21, 17.11] | | **0.04*** |
| | RCT: 1 | FNS vs CCS | -2.63 [-9.02-, 3.76] | 92.9% | 0.42 |
| | | DHS vs FNS | 11.29 [1.80, 20.79] | | **0.02*** |
| **Follow-up duration** | | | | | |
| Harris Hip Score | **≥6mo: 3** | DHS vs CCS | 0.54 [-6.04, 7.13] | | 0.87 |
| | | FNS vs CCS | 3.26 [-0.72, 7.24] | 83.5% | 0.11 |
| | | DHS vs FNS | -2.72 [-9.24, 3.81] | | 0.41 |
| | **≥12mo: 12** | DHS vs CCS | 2.93 [-0.82, 6.69] | | 0.13 |
| | | FNS vs CCS | 3.9573 [1.00, 6.91] | 91.2% | **0.01*** |
| | | DHS vs FNS | -1.02 [-5.33, 3.29] | | 0.64 |

## The results of meta-regression based on age.

**Table S4. The results of meta-regression based on age.**

| Outcomes | Treatment comparisons | Mean(95%CI) | Regression Coefficient (B)(95%CI) | Standardized Age (Mean±SD) | $I^2$ | DIC |
|---|---|---|---|---|---|---|
| HHS | DHS vs CCS | 2.54 [-1.23, 6.21] | -4.57 [-10.28, 0.62] | 55.46±23.66 | 10% | 61.8 |
| | FNS vs CCS | 3.76 [0.99, 6.60] | | | | |
| Intraoperative blood loss(ml) | DHS vs CCS | 12.74 [1.22, 24.48] | -15.15 [-30.72, 0.98] | 55.80±17.87 | 7% | 70.58 |
| | FNS vs CCS | -0.50 [-10.07, 8.89] | | | | |
| Operative time(min) | DHS vs CCS | 51.80 [18.40, 85.65] | -45.95 [-91.99, -2.61] | 52.77±11.58 | 5% | 45.86 |
| | FNS vs CCS | 35.68 [10.44, 61.50] | | | | |

# Table S5: CINeMA Confidence Ratings for Primary and Secondary outcomes

**Table S5 (part 1 of 3): CINeMA Confidence Ratings for Primary and Secondary outcomes.**

| Comparison | Number of studies | Within-study bias | Reporting bias | Indirectness | Imprecision | Heterogeneity | Incoherence | Confidence rating | Reason(s) for downgrading |
|---|---|---|---|---|---|---|---|---|---|
| **Harris Hip Score** | | | | | | | | | |
| CCS vs DHS | 6 | Some concerns | Low risk | No concerns | Some concerns | Some concerns | No concerns | Low | ①④⑤ |
| CCS vs FNS | 9 | Some concerns | Low risk | No concerns | No concerns | Some concerns | No concerns | Low | ①⑤ |
| DHS vs FNS | 2 | Some concerns | Low risk | No concerns | No concerns | Major concerns | No concerns | Very low | ①⑤ |
| **Femoral head necrosis** | | | | | | | | | |
| CCS vs DHS | 11 | Some concerns | Low risk | Some concerns | No concerns | Some concerns | No concerns | Low | ①③⑤ |
| CCS vs FNS | 9 | Some concerns | Low risk | No concerns | Some concerns | No concerns | No concerns | Low | ①④ |
| DHS vs FNS | 4 | Some concerns | Low risk | No concerns | No concerns | No concerns | No concerns | Moderate | ① |

Note: ①=Within-study bias; ②=Reporting bias; ③=Indirectness; ④=Imprecision; ⑤=Heterogeneity; ⑥=Incoherence

**Table S5 (part 2 of 3): CINeMA Confidence Ratings for Primary and Secondary outcomes.**

| Comparison | Number of studies | Within-study bias | Reporting bias | Indirectness | Imprecision | Heterogeneity | Incoherence | Confidence rating | Reason(s) for downgrading |
|---|---|---|---|---|---|---|---|---|---|
| **Femoral neck shortening** | | | | | | | | | |
| CCS vs DHS | 1 | Some concerns | Low risk | No concerns | Major concerns | No concerns | No concerns | Very low | ①④ |
| CCS vs FNS | 3 | Some concerns | Low risk | No concerns | Major concerns | No concerns | No concerns | Very low | ①④ |
| DHS vs FNS | 1 | Some concerns | Low risk | No concerns | Major concerns | No concerns | No concerns | Very low | ①④ |
| **Implant failure/cut-out** | | | | | | | | | |
| CCS vs DHS | 8 | Some concerns | Low risk | No concerns | No concerns | Major concerns | No concerns | Very low | ①⑤ |
| CCS vs FNS | 9 | Some concerns | Low risk | No concerns | Some concerns | Some concerns | No concerns | Low | ①④⑤ |
| DHS vs FNS | 4 | Some concerns | Low risk | No concerns | Major concerns | No concerns | No concerns | Very low | ①④ |
| **Fracture nonunion/delayed union** | | | | | | | | | |
| CCS vs DHS | 10 | Major concerns | Low risk | Major concerns | No concerns | No concerns | No concerns | Very low | ①③ |
| CCS vs FNS | 8 | Some concerns | Low risk | No concerns | Some concerns | No concerns | No concerns | Low | ①④ |
| DHS vs FNS | 3 | Some concerns | Low risk | Some concerns | Some concerns | No concerns | No concerns | Low | ①③④ |

Note: ①=Within-study bias; ②=Reporting bias; ③=Indirectness; ④=Imprecision; ⑤=Heterogeneity; ⑥=Incoherence

**Table S5 (part 3 of 3): CINeMA Confidence Ratings for Primary and Secondary outcomes.**

| Comparison | Number of studies | Within-study bias | Reporting bias | Indirectness | Imprecision | Heterogeneity | Incoherence | Confidence rating | Reason(s) for downgrading |
|---|---|---|---|---|---|---|---|---|---|
| **Intraoperative blood loss** | | | | | | | | | |
| CCS vs DHS | 3 | Some concerns | Low risk | No concerns | No concerns | No concerns | No concerns | Moderate | ① |
| CCS vs FNS | 7 | Some concerns | Low risk | No concerns | No concerns | No concerns | No concerns | Moderate | ① |
| DHS vs FNS | 3 | Some concerns | Low risk | No concerns | No concerns | No concerns | No concerns | Moderate | ① |
| **Operative time** | | | | | | | | | |
| CCS vs DHS | 6 | Some concerns | Low risk | No concerns | No concerns | Some concerns | No concerns | Low | ①⑤ |
| CCS vs FNS | 10 | Some concerns | Low risk | No concerns | No concerns | Major concerns | No concerns | Very low | ①⑤ |
| DHS vs FNS | 3 | Some concerns | Low risk | No concerns | No concerns | Some concerns | No concerns | Low | ①⑤ |
| **Fracture healing time** | | | | | | | | | |
| CCS vs DHS | 1 | Some concerns | Low risk | Some concerns | Some concerns | Some concerns | No concerns | Very low | ①③④⑤ |
| CCS vs FNS | 5 | Some concerns | Low risk | No concerns | No concerns | Some concerns | No concerns | Low | ①⑤ |
| DHS vs FNS | 1 | Some concerns | Low risk | No concerns | Some concerns | Some concerns | No concerns | Low | ①④⑤ |

Note: ①=Within-study bias; ②=Reporting bias; ③=Indirectness; ④=Imprecision; ⑤=Heterogeneity; ⑥=Incoherence
