# Supplementary material for: Comparative efficacy and safety of three internal fixation strategies for femoral neck fractures: a network meta-analysis
Source: Front Med (Lausanne). 2026 Mar 2;13:1782357. doi: 10.3389/fmed.2026.1782357 (PMC12989485; doi:10.3389/fmed.2026.1782357)
Supplement: Supplementary file 2 [file Table_1.DOCX]

**Table 1 (part 1 of 2): Characteristics of the included studies.**

| **Study** | **Design** | **Country** | **Type/classification of fractures** | **Operation** | **Number of patients** | **Age(year)** | **Sex (Male)** | **Follow-up term(month)** | **Outcomes** |
| --- | --- | --- | --- | --- | --- | --- | --- | --- | --- |
| Lee YS et al. 2008 | CS | China, Taiwan | Garden I-II, Pauwels I-III | CCS | 32 | 72.8±3.9 | 17 | ≥12 | 1,2,4,5,7,8 |
|  |  |  |  | DHS | 25 | 74.6±4.1 | 12 |  |  |
| Watson A et al. 2013 | RCT | Australia | Garden I-II | CCS | 29 | 76.7 | 5 | 24 | 1,2,4,5 |
|  |  |  |  | DHS | 31 | 77.9 | 6 |  |  |
| Siavashi B et al. 2015 | RCT | Iran | Garden III-IV | CCS | 28 | 28 | 21 | ≥12 | 1,2,4 |
|  |  |  |  | DHS | 30 | 30 | 25 |  |  |
| Gupta M et al. 2016 | RCT | India | Garden I-IV | CCS | 45 | 39.3 | 32 | 18-48 | 1,2,4,5,6,7 |
|  |  |  |  | DHS | 40 | 40.7 | 23 |  |  |
| Jettoo P et al. 2016 | CS | UK | NR | CCS | 34870 | ≥50 | 9236 | 48 | 2,5 |
|  |  |  |  | DHS | 18014 | ≥50 | 4671 |  |  |
| Chen C et al. 2017 | CS | China | Garden II-IV | CCS | 44 | 56.8±9.3 | 20 | ≥24 | 1,2,3,4,5,6,7 |
|  |  |  |  | DHS | 42 | 58.3±8.6 | 18 |  |  |
| Aaron N et al. 2017 | RCT | Canada | Garden I-IV, Pauwels I-III | CCS | 537 | 72.0±12.3 | 210 | 24 | 2,3,4,5 |
|  |  |  |  | DHS | 542 | 72.2±12.0 | 212 |  |  |
| Şahin A et al. 2020 | CS | Turkey | Garden I-IV | CCS | 37 | 41.9±13.9 | 27 | 12-36 | 1,2,3,4,5,7 |
|  |  |  |  | DHS | 41 | 45.7±12.6 | 30 |  |  |
| Hu H et al. 2021 | CS | China | Garden I-IV, Pauwels I-III | CCS | 24 | 50.46±9.26 | 14 | ≥12 | 1,2,3,4,5,6,7,8 |
|  |  |  |  | FNS | 20 | 50.45±8.45 | 12 |  |  |
| Tang Y et al. 2021 | CS | China | Garden II-IV, Pauwels I-III | CCS | 45 | 54.8±11.7 | 13 | 14-24 | 1,2,3,4,5,6,7,8 |
|  |  |  |  | FNS | 47 | 57.4±15.0 | 34 |  |  |
| Zhou XQ et al. 2021 | CS | China | Pauwels III | CCS | 30 | 53.14±7.19 | 12 | 10-22 | 1,2,3,4,5,6,7 |
|  |  |  |  | FNS | 30 | 54.53±6.71 | 12 |  |  |
| Zhang YZ et al. 2022 | CS | China | Garden II-IV | CCS | 36 | 52±10.72 | 21 | ≥6 | 1,3,7 |
|  |  |  |  | FNS | 33 | 57.61±11.87 | 22 |  |  |

**Table 1 (part 2 of 2): Characteristics of the included studies.**

| **Study** | **Design** | **Country** | **Type/classification of fractures** | **Operation** | **Number of patients** | **Age(year)** | **Sex (Male)** | **Follow-up term(month)** | **Outcomes** |
| --- | --- | --- | --- | --- | --- | --- | --- | --- | --- |
| Abdallatif AG et al. 2023 | CS | UK | Garden I-II、Pauwels I-III | CCS | 68 | 77.28±8.16 | 60 | ≥24 | 2,5 |
|  |  |  |  | DHS | 80 | 79.56±8.93 |  |  |  |
| Ge Z et al. 2023 | CS | China | Garden II-IV, Pauwels I-III | FNS | 43 | 48.2±8.0 | 30 | ≥18 | 1,2,3,4,5,6,7 |
|  |  |  |  | DHS | 52 | 50.4±7.4 | 37 |  |  |
| Kenmegne GR et al. 2023 | CS | China | Garden I-IV, Pauwels I-III | CCS | 58 | 40.45±16.5 | 34 | 12-36 | 1,2,3,4,5,6,7,8 |
|  |  |  |  | FNS | 56 | 58.2±15.15 | 29 |  |  |
| Niemann M et al. 2023 | CS | Germany | NA | FNS | 23 | 66(57,75) | 24 | ≥6 | 2,3,4 |
|  |  |  |  | DHS | 23 |  |  |  |  |
| Xu X et al. 2023 | CS | China | Garden I-IV, Pauwels I-III | CCS | 51 | 61.6±16.4 | 17 | ≥9 | 1,2,3,4,5,6,7 |
|  |  |  |  | FNS | 54 | 60.7±15.2 | 18 |  |  |
|  |  |  |  | DHS | 52 | 63.1±13.2 | 14 |  |  |
| Yan SG et al. 2023 | CS | China | Garden I-IV, Pauwels I-III | CCS | 27 | 46.15±11.76 | 14 | ≥12 | 1,2,3,4,5,6,7,8 |
|  |  |  |  | FNS | 22 | 51±7.11 | 12 |  |  |
| Bukhary HA et al. 2024 | CS | Kingdom of Saudi Arabia | Garden I-II | CCS | 41 | 70.6±7.6 | 35 | ≥12 | 2,5,7 |
|  |  |  |  | DHS | 44 | 72±5.4 |  |  |  |
| Caldaria A et al. 2024 | CS | Italy | Garden I-IV, Pauwels I-III | CCS | 74 | 68.9 | 18 | ≥12 | 1,2,4,7 |
|  |  |  |  | FNS | 70 | 66.3 | 26 |  |  |
| Chung H et al. 2024 | CS | Korea | Garden I-IV, Pauwels I-III | CCS | 65 | 55.9±15 | 28 | ≥6 | 2,4,5,7,8 |
|  |  |  |  | FNS | 40 | 58.7±10.5 | 19 |  |  |
| Hong H et al. 2024 | RCT | China | Garden II-IV | FNS | 45 | 55.19±10.01 | 25 | ≥6 | 2,4,5,6,7,8 |
|  |  |  |  | DHS | 45 | 54.42±9.62 | 26 |  |  |
| Zheng S et al. 2024 | CS | China | Garden III-IV | CCS | 90 | 52(45.8,56) | 50 | ≥18 | 1,2,3,4,5,6,7 |
|  |  |  |  | FNS | 135 | 51(42,57) | 85 |  |  |

**Table 1:**CS=Cohort study; RCT=Randomized controlled trial; CCS=Cannulated compression screw; DHS=Dynamic hip screw; FNS=Femoral neck system; NA=Not applicable. Outcomes:1=Harris Hip Score,2=femoral head necrosis,3=Femoral neck shortening,4=Implant failure/cutout,5=Fracture nonunion/delayed union, 6=Intraoperative blood loss, 7=Operation time,8=Fracture healing time.
